# Unravelling the Nexus of Beach Litter and Plant Species and Communities Along the Mediterranean Coasts: A Critical Literature Review

**DOI:** 10.3390/plants13223125

**Published:** 2024-11-06

**Authors:** Giulia Calderisi, Donatella Cogoni, Giuseppe Fenu

**Affiliations:** Department of Life and Environmental Sciences, University of Cagliari, Viale Sant’Ignazio da Laconi 13, 09123 Cagliari, Italy; giulia.calderisi@unica.it (G.C.); d.cogoni@unica.it (D.C.)

**Keywords:** coastal dunes, environmental pollution, marine beach litter, Mediterranean Basin, methodological approaches, psammophilous ecosystems

## Abstract

Beach litter, an anthropogenic and hazardous component, can interact with psammophilous plant species and communities. These are particularly prominent in the Mediterranean Basin, renowned for its highly specialized and unique flora but recognized as one of the areas that is globally most severely affected by marine litter. To provide a comprehensive picture and outline possible future directions, data on beach litter in the Mediterranean coastal ecosystems were collected through a bibliographic research. Overall, 103 studies investigated the presence of beach litter on the Mediterranean coasts, of which only 18 considered its relationship with psammophilous plant species and communities. Our research highlights that this topic is rather underexplored in the Mediterranean Basin and the need to develop a standardized protocol for the assessment of beach litter that can be applied consistently across different beaches and countries. Information collected through a standardized protocol might improve the management and conservation strategies for these fragile ecosystems.

## 1. Introduction

Marine litter represents an escalating global concern, with its often disregarded consequences posing threats to coastal, freshwater, and marine ecosystems [1,2,3]. According to the United Nations Environment Programme (UNEP) [4], marine litter is defined as “any persistent, manufactured, or processed solid material discarded, disposed of, or abandoned in the marine and coastal environment”. Closely connected to this issue is beach litter, which comprises the portion of marine litter that is washed ashore by wind, sea currents, and waves [5]. This type of litter can also originate from the mainland. Consequently, coastal areas have become hotspots for litter accumulation [6,7,8], making beach litter one of the most visible indicators of this environmental pollution issue. Marine litter, particularly plastic waste, is a consequence of human pollution [9]. The presence of this component is primarily attributed to the inadequate management of urban and industrial solid waste, the discharge of untreated or improperly treated wastewater, agricultural practices, coastal tourism, and recreational activities [10]. Sea-based sources encompass fishing (vessels, angling, and fish farming), shipping (merchant and public transport; pleasure, naval, and research vessels), offshore mining and extraction (vessels and oil and gas platforms), and natural disasters [4,10]. The interaction between sea-based (shipping) and land-based (human use) human activities is fundamental among the anthropic factors and significantly influences the number and composition of objects found on the beach [11]. Furthermore, natural elements like winds, currents, tides, waves, river flows, canals, drains, sewage outlets, and stormwater discharge facilitate the entry of marine debris into coastal and marine environments [12,13]. These natural factors can cause the transport of litter over long distances before being deposited on the shorelines or on the ocean floor. This suggests that the beach litter discovered at a particular location may be native to the area, may have arrived from the inland, or may have traveled great distances through prevailing winds and ocean currents [14]. For these reasons, identifying the sources of litter can often be challenging, especially for items that have been in the marine environment for a long time [15].

Marine litter is ubiquitous across all the world’s oceans, reaching far beyond densely populated regions into remote areas untouched by human presence or obvious sources [4]. Although there is a wide variety of litter materials in marine environments, plastic is the most commonly recorded material [16,17]. In particular, despite ongoing initiatives and efforts, the amount of plastic in the oceans is estimated to range from 75 to 199 million tons, and it is increasing exponentially over time [13]. This type of litter is extremely hazardous both for humans and animals. For example, it can lead to death or can induce sub-lethal consequences (e.g., entanglement, laceration of internal tissue, physiological stress, and toxicological harm) in various marine animals, including whales, seals, turtles, birds, and fish, as well as invertebrates like bivalves, plankton, worms, and corals [13]. The detrimental impact of marine debris on animal species, particularly through entanglement and ingestion, has been investigated in numerous studies, e.g., [18,19,20,21,22]. In particular, microplastics, which are particles measuring millimeters or smaller, have become a focal point of recent environmental concern due to their status as the most prevalent and potentially hazardous component of litter in the ocean [23]. The widespread presence of microplastics has been documented across various marine habitats, including surface and subsurface water columns, e.g., [24,25]; beach and wetland sediments [26]; polar regions, e.g., [27,28]; and even the deep sea, e.g., [29,30]. Once ingested, microplastics can translocate within the tissue of different organs, be egested via pseudo-feces, or accumulate in specific body tissue types [31,32]. When microplastics are embedded in body tissue, they can cause multiple detrimental effects on the organism’s health, including infertility, inhibited growth, internal or external injuries, and the obstruction of bodily passages, among others [33,34,35]. Furthermore, with the escalating proliferation of plastic litter, the infiltration of micro- and nanoplastics into the ecological trophic chain poses a significant hazard to human health [36,37,38]. The ingress of micro- and nanoplastics into the human trophic chain has been reported in several research studies, highlighting that this can occur via various pathways: ingestion by animals in their natural habitats [39], contamination during food production processes [40], and/or leaching from plastic packaging used for food and beverages [41]. To date, micro- and nanoplastic fragments have been observed in diverse consumables, such as honey, beer, energy drinks, soft drinks, salt, sugar, fish, shrimps, and bivalves, e.g., [42,43,44,45,46]. Specifically, the ingestion of microplastics through seafood consumption by human populations residing in coastal and indigenous communities, where marine species constitute the primary food source, is likely to pose significant threats [13]. Moreover, microplastics have the potential to ingress into the human body through inhalation and dermal absorption, accumulating within organs, including the placenta [13,47].

Nevertheless, despite considerable research efforts aimed at assessing the impact of marine litter on marine biota and ecosystems, only a limited body of literature has concentrated on coastal dune plants and habitats. Despite this, beach litter can interact in different ways with the psammophilous plant species and communities characteristic of these ecosystems; in fact, psammophilous plants can be considered focal points for beach litter accumulation [48]. In recent years, some studies have reported the ability of psammophilous plant species and communities in coastal dunes to trap beach litter, e.g., [49,50,51,52,53,54,55,56,57]. Other studies have assessed how the distribution of beach litter varies in different dune habitats [1,52,58,59,60]. In addition, seed germination studies were carried out to investigate the effects of some beach litter categories on seed germination and on the vegetative propagules of specific dune plants [61,62]. Psammophilous plants are a key element in coastal ecosystems due to their pioneering ability to colonize bare sands. These plants are ecologically important as they act as ecosystem engineers, stabilizing the sediment and shaping the morphologies of coastal dunes.

Coastal dunes are unique and dynamic ecosystems with significant ecological value and high biodiversity; they represent an essential environmental resource with a variety of ecosystem functions, products, and services, such as shoreline protection, water filtration, and nutrient cycling, e.g., [63,64,65,66,67,68,69,70,71,72]. These unique ecosystems are threatened by a variety of factors, including climate change, biological invasion, urbanization, misuse (e.g., mechanical beach cleaning), tourism, and various pollution types [70,73,74,75,76,77,78,79]. These threats can cause habitat loss, fragmentation, and coastal erosion, resulting in the complete and sometimes permanent destruction of coastal dune systems [80,81]. In particular, among these different threats, beach litter appears to have a global distribution range [82]. In the last few years, it has been demonstrated that beach litter accumulation could exert selective pressure on all components of biodiversity and related ecosystem services across a broad spectrum of ecological domains, including biological populations and communities and biogeochemical cycles [83,84,85]. For example, litter can serve as a vector for the spread of invasive species, affecting or modifying species assemblages [86,87], or as carriers of chemicals and contaminants, e.g., [36].

The plant species and communities present in dune systems are notably expressed in the Mediterranean Basin, a semi-enclosed basin that is renowned for its highly specialized and distinctive flora [66,88,89,90]. The Mediterranean Basin is recognized as a priority center of plant diversity. Although it constitutes merely 1.6% of the Earth’s surface, this region harbors approximately 7% of the global plant species; for this reason, it has been recognized as one of the world’s largest hotspots of biodiversity [90,91,92]. However, the distribution of plant richness is uneven [93]. Notably, plant diversity is found on the major Mediterranean islands, including Sicily, Sardinia, Cyprus, Corsica, and Crete, as well as in the archipelagos, such as the Balearic, Tuscan, and Aeolian islands, which exhibit an endemism rate exceeding 40% [90,92]. The distinctive insular characteristics of the Mediterranean, such as the different geographical situations, as well as their geographical and paleogeographic evolution, contribute to unique patterns of plant diversity and associations; as a result, the islands and islets (and archipelagos) within the Mediterranean Basin exhibit exceptional floral diversity and serve as primary centers of plant diversity, particularly due to the limited distribution of many plant species [90,91,92]. At the same time, the Mediterranean Sea has been identified as one of the regions most profoundly influenced by marine litter on a global scale [94]. Indeed, the aggregate accumulation of plastic in the Mediterranean Sea is estimated to be in the order of magnitude of 1,178,000 tons, with an uncertainty range spanning from 53,500 to 3,546,700 tons [95], thereby positioning it as a region profoundly impacted by plastic pollution [95,96,97,98,99]. From this perspective, beach litter should be considered a serious concern for Mediterranean coastal ecosystems, necessitating the monitoring of this anthropic component, especially in relation to the presence of psammophilous plant species and communities, and the implementation of initiatives to limit the occurrence of this component in the coastal dune systems. To achieve these objectives, it would be suitable to establish procedures that can be used at an international level, allowing for comparable data across countries and filling an important gap in the Mediterranean Basin at the biogeographical level. In fact, in the Mediterranean Basin, there has been an increase in knowledge about the distribution, amount, and type of litter, followed by an increase in the sampling of offshore areas [100]. However, this has not always been accompanied by equally increasing interest in the standardization of the protocols and procedures used to gather solid and comparable data on the amount, composition, and distribution of beach litter [14,100].

In this context, the current study aims to comprehensively evaluate the literature pertaining to the Mediterranean Basin, focusing on the correlation between beach litter and psammophilous plant species and communities. To achieve this overall aim, three specific objectives are outlined: (1) to analyze papers on marine litter in the Mediterranean Basin, selecting those focused on beach litter in coastal dune systems; (2) to evaluate the methodologies employed to investigate the presence and distribution of beach litter in Mediterranean dune systems; and (3) to assess the specific methodologies utilized in examining the impact of beach litter on psammophilous plant species and communities.

## 2. General Overview of Beach Litter’s Presence in the Mediterranean Basin

After merging the results of each search and removing duplicates, a total of 1136 articles published between 1986 and 2023 were identified. Additionally, 65 articles relevant to the research topic, which were discovered during the literature review process, were manually included, bringing the total number of articles to 1201.

In a subsequent step, a total of 965 articles were excluded as they addressed topics divergent from our research focus or were conducted outside the Mediterranean Basin. The remaining 236 articles were then categorized based on the ecosystem considered as the study area. Among these articles, some focused on multiple ecosystems. In particular, the three categories identified were (1) “coastal ecosystems”, encompassing articles with coastal ecosystems as study areas (103 articles, equal to 37.73%); (2) “marine ecosystems”, including articles investigating marine ecosystems (138 articles, equal to 50.55%); and (3) “freshwater ecosystems”, comprising articles examining rivers, lakes, and lagoons as study areas (32 articles, equal to 11.72%). It is interesting to note that, although keywords related to beach litter were used, a considerable number of publications focused on marine ecosystems. This highlights a dearth of research specifically addressing coastal ecosystems in comparison to marine ones.

**Figure 1:**
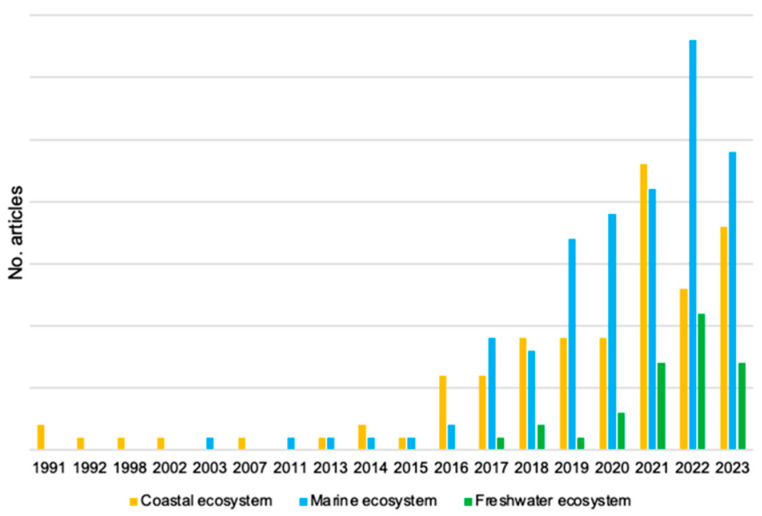
Temporal trend in scientific studies dealing with litter in coastal ecosystems (yellow), marine ecosystems (blue), and freshwater ecosystems (green).

Analyzing the temporal distribution of the articles across these three categories, it can be noted that the interest in this topic has seen an exponential increase since 2017 (Figure 1).

Focusing on the “coastal ecosystem” category, only a limited number of articles were published between 1991 and 2015 (one or two per year). Moreover, during certain years within this timeframe, no articles related to the research topic were published. Interest in the role of beach litter in coastal ecosystems has increased notably only since 2016 (Figure 1, Table S1). This interest has markedly intensified over the past three years, reaching its peak in 2021, with the publication of the largest number of studies (23 articles).

The increase in the interest in investigating beach litter in coastal ecosystems, especially in recent years, could be attributed to several factors. Firstly, it is necessary to consider the environmental impact that this litter has on ecosystems, exerting selective pressure on all aspects of biodiversity and related ecosystem services [83,84,85]. Another important factor could be the growth in public awareness, as well as the release of guidelines for the monitoring of marine litter on beaches, e.g., [94,101,102,103].

## 3. General Methodological Approaches Used to Evaluate Beach Litter on the Mediterranean Sandy Coasts

**Figure 2:**
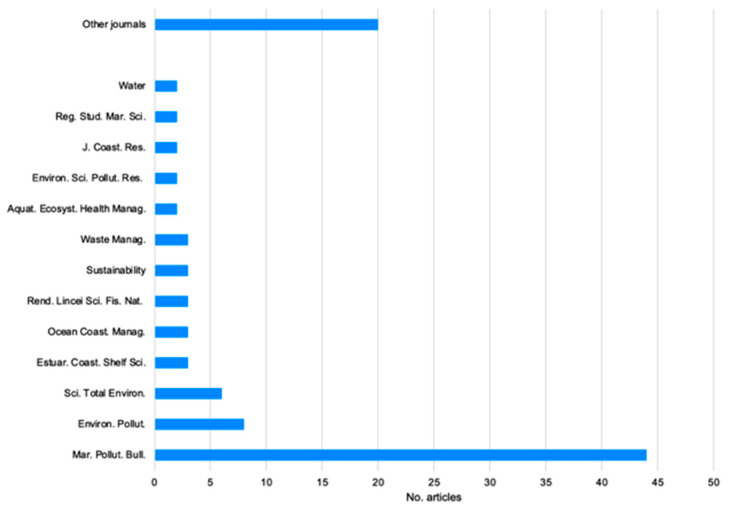
Number of articles about beach litter in the coastal environment, relating to the Mediterranean Basin, divided according to the different journals. Other journals include *Aquatic Toxicology*; *Environmental Footprints and Eco-Design of Products and Processes*; *Environmental Monitoring and Assessment*; *Environmental Practice*; *Environments* (MDPI); *Frontiers in Marine Science*; *Geo-Eco-Marina*; *Handbook of Microplastics in the Environment*; *Journal of Analytical Chemistry*; *Journal of Insect Conservation*; *Land*; *Marine Environmental Research*; *Marine Policy*; *Mediterranean Marine Science*; *Plastic Pollution and Marine Conservation*; *Scientia Marina*; *Scientific Reports*; *The Montenegrin Adriatic Coast*; *The Handbook of Environmental Chemistry*; *Turkish Journal of Fisheries and Aquatic Sciences*; and *Water, Air, & Soil Pollution*.

Focusing uniquely on the “coastal ecosystem” category, which comprises 103 articles, the majority of these were published in the *Marine Pollution Bulletin* journal (42.72%). Additionally, some articles were published in the *Environmental Pollution* journal (7.77%) and in the *Science of the Total Environment* journal (5.83%; Figure 2). The remaining journals each had a relatively small number of articles; in particular, in many of them (19.42%), only one article related to the research topic was published (Figure 2).

Only a few journals address the underlying concerns associated with the presence of beach litter on coastlines and the threat that it poses to psammophilous communities and dune habitats. To facilitate the greater dissemination of information, it would be necessary for a larger number of journals to publish articles relating to this topic.

**Figure 3:**
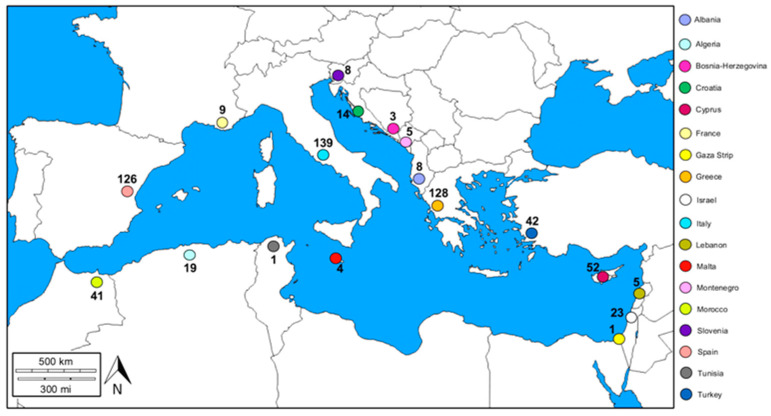
Locations and number of beaches, for each country in the Mediterranean Basin, in which the presence of beach litter has been analyzed.

Based on our results, it is evident that, at the Mediterranean Basin level, the distribution of studies aimed at investigating the presence and related impacts of beach litter on coastal dune systems is not homogeneous. The geographical areas in which the samplings were carried out and the number of beaches considered for each area are represented in Figure 3. The country with the largest number of investigated beaches was Italy (22.13%), followed by Greece (20.38%) and Spain (20.06%). A fairly large number of sampling beaches was also found in Cyprus (8.28%), Turkey (6.69%), and Morocco (6.53%). Meanwhile, a small number of sampled beaches was found in the following countries: Lebanon and Montenegro (0.80% for each country), Malta (0.64%), and Bosnia-Herzegovina (0.48%). Of the 628 beaches identified through bibliographic research, it was possible to precisely locate 618. Among these, the majority were situated in continental contexts (61.33%), while the remaining beaches were found in island contexts (38.67%). Specifically, 57.32% of the beaches in island contexts were situated on large islands (such as Sardinia, Sicily, Corsica, Cyprus, Crete, and Mallorca), while the remaining 42.68% were associated with beaches on small islands (e.g., Malta, Menorca, and Ibiza).

Considering the distribution of articles among Mediterranean countries, Italy emerged with the largest number of articles (with 40 articles, 34.78%), followed by Spain (with 13 articles, 11.30%) and Greece (with 11 articles, 9.57%). Both Cyprus and Israel were the focus of seven articles each (6.09% for each country). Morocco and Turkey were each considered in six papers (5.22% for each), while Croatia was analyzed in five papers (4.35%). Furthermore, three articles each were dedicated to the exploration of beach litter in France and Montenegro (each 2.61%). Regarding Albania, Algeria, Bosnia, Lebanon, Malta, and Slovenia, the number of articles was equal to two for each country (1.74% for each country), and only one article took both the Gaza Strip and Tunisia into consideration (0.87% for each).

In certain countries, many beaches have been investigated (e.g., Italy, Greece, Spain, Cyprus). However, there are other regions where beach investigations remain limited, such as France, Albania, Slovenia, and Lebanon, or entirely unexplored, including Tunisia, the Gaza Strip, Egypt, Syria, Libya, and the Strait of Gibraltar. Remarkably, despite the abundance of beaches in the Southern Mediterranean Basin, there is a low level of research activity inherent to the study of beach litter in these areas. This was also reported by Abidli et al. [104], who highlighted the insufficient analysis of plastic pollution along the Southern Mediterranean coastlines. The reduced number of studies in these areas could be due to several factors. An example could be a lack of awareness of the relationship between dune systems and beach litter, which leads these countries to direct research towards other areas deemed more urgent. Another reason could be related to the difficulty in reaching the beaches. In fact, in some areas on the southern coast of the Mediterranean Basin, the beaches may be in remote or difficult-to-access places due to the geography of the area. Alternatively, the shortage of data may be due to political and security instabilities, as is the case in some areas of Libya, Syria, and the Gaza Strip. Moreover, this lack of data could have been derived from a limitation in the bibliographic search itself, i.e., using the keywords reported in the Materials and Methods section did not highlight all published works. Furthermore, it is possible that additional information exists in non-indexed publications or in the grey literature for some Southern Mediterranean countries. These sources are often published in local languages, making them inaccessible to an international audience or difficult to find through the most common scientific databases. Nevertheless, to obtain an overall picture of the current impact of beach litter in the dune systems of the Mediterranean Basin, it would be necessary to increase the number of studies in countries where the topic is rarely or not investigated at all.

It must also be considered that characterizing beach litter at the single beach level can provide limited information regarding the actual amount of litter produced by a country and its sources. Since the Mediterranean Basin is a semi-enclosed system, litter originating from one country can easily reach another through sea currents and winds. In fact, it is common to find litter washed up on the shoreline of a particular beach, with labels or identifiable information that links it to a different country. Therefore, given that understanding the sources of litter is crucial for planning actions to clean up litter and prevent its disposal and accumulation [58], it is necessary to pay closer attention to these variables.

The methodologies used in the field to analyze the beach litter appear to be considerably different country by country. Considering the 103 total articles, 15 of these were reviews and one was a descriptive study. The remaining 87 articles were based on data collected in the field, using different sampling systems.

**Figure 4:**
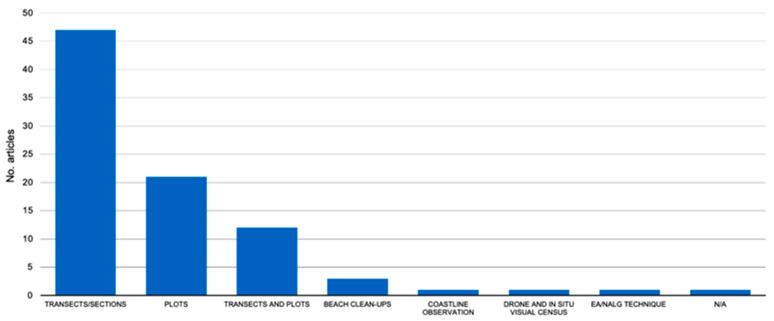
Number of articles for each methodology identified for the study of beach litter in Mediterranean coastal dune systems. N/A = data not indicated.

The main methods used to investigate beach litter were the use of transects/sections (54.02%) and of plots (24.14%; Figure 4). In some articles, the simultaneous use of transects and plots for beach litter analysis has been reported (13.79%). Additional methodologies (e.g., beach clean-ups, coastline observations, the use of drones) have been employed but are reported in a very small number of articles (Figure 4).

The dimensions chosen for the transects/sections and plots vary across different articles (Table S1). Some articles specify the use of 100-m transects/sections, e.g., [105,106,107,108,109,110,111], while others use 50-m-long transects/sections, e.g., [112,113,114]. Additionally, some studies employ transects/sections of different sizes, such as those extending from the waterline to the back of the beach, e.g., [115,116,117,118], or transects that are 80 or 90 m long [119,120] (Table S1). Furthermore, certain articles report the use of transects/sections with dimensions tailored to the specific sizes of the beaches, e.g., [121,122,123,124] (Table S1). The choice to use transects of 100 or 50 m in length is based on European technical manuals, e.g., [5,101,102,125], which define these as optimal units of measurement for the study of beach litter. Conversely, the use of transects/sections of varying sizes, specific to each beach analyzed, stems from an expert-based method. In this method, the author independently determines the ideal sampling size for the particular beach.

The size of the plots also varies, particularly depending on the aims of the study and the type of beach litter under investigation. For example, some studies used 4 m^2^ plots, e.g., [1,58,59,126], while others reported the use of 1 m^2^ plots, e.g., [51,52,127] (Table S1). The study of beach litter’s presence and impacts in dune systems is mainly based on comparisons among various beaches in different study areas. It should be noted that the choice of different sampling systems, each with different dimensions, complicates the comparison of data across different studies. However, given the considerable variability in dune systems, particularly in depth and extent, it is necessary to acknowledge that the use of standardized procedures is not always possible.

In addition, the number of surveys conducted at each beach varies from article to article. Most articles report that surveys were repeated multiple times during the monitoring period (24.72%). In many cases, measurements were conducted only once (22.47%), while, in others, this information was not provided (21.35%). Some articles reported the biannual monitoring of the beaches (10.11%), others reported seasonal monitoring (12.36%), and some reported monthly monitoring (8.99%; Table S1). To our knowledge, there is no standardized number of surveys that must be conducted on a specific beach to effectively evaluate the presence and impact of beach litter. The number of surveys required may vary depending on the specific aims of the study. For instance, if the aim is to obtain information on the presence of beach litter, a single survey may suffice. However, if the aim is to study the accumulation and distribution of beach litter, it is necessary to implement procedures with repeated, well-defined sampling over a certain period, such as monthly, quarterly, or annually [102]. The European manual proposed by Galgani et al. [101] recommends at least four surveys per year in spring, summer, autumn, and winter. Preferably, these surveys should be repeated on the same day each year. Furthermore, it is recommended to record the coordinates of the sampling points to ensure the repeatability and accuracy of the survey [101,128]. The “African Marine Litter Monitoring Manual” [129] states that standing-stock surveys should be conducted once (e.g., over the course of a single day) to quickly evaluate the quantity and types of litter present on a beach at a specific moment. To conduct accumulation surveys, the site must first undergo a clean-up on day zero, removing all visible litter from a designated section of the beach, followed by daily clean-ups for ten consecutive days. Each day, the accumulated litter is collected, cleaned, classified into predefined categories, and then weighed and counted [129].

The strategies used to evaluate beach litter in the different studies were also considerably different. The first distinction lies in the methods of collection and classification of beach litter. Among the surveyed works, 67 specified that litter was collected in the field, while 13 did not conduct beach litter collection, and seven lacked information on the collection methods. Researchers should consider the collection of beach litter from dune systems, as it can enhance the accuracy of data reporting and reduce the impact of the litter on these ecosystems [14,101]. However, it is necessary to consider that this is not always possible. Depending on the research objectives (e.g., a temporal analysis aimed at studying how the distribution of waste changes over time) and methodology (e.g., coastline observation of remote beaches or use of drones), for researchers, it may be necessary to leave the beach litter in the dune system.

Regarding the classification of beach litter into different categories, this was performed in 83 studies; in two articles, no classification was described, and two articles lacked this information (Table S1). The classification process is crucial, as it provides information on the composition of beach litter and offers details about specific litter items and their sources [58]. Based on our results, this classification is performed by applying different protocols or by dividing the litter into categories, which are chosen by the researcher based on their research objectives. Moreover, in this case, it would be necessary to identify a single classification method that allows the easier comparison of data from different beaches and countries.

**Figure 5:**
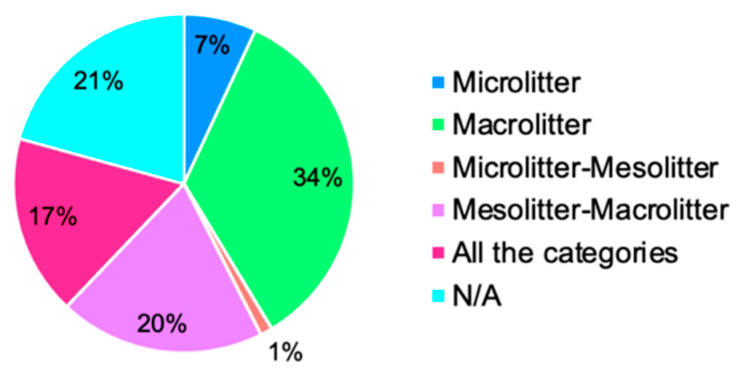
Percentage of studies that investigated the different categories of beach litter. All categories = microlitter, items up to 5 mm in the longest dimension; mesolitter, items from 5 mm to 25 mm in the longest dimension; and macrolitter, items above 25 mm in the longest dimension [103]; N/A = data not indicated.

The analysis of the dimensions of the beach litter investigated provides further interesting elements for discussion. In the articles considered, this information was reported in the form of numerical values (e.g., >2.5; <0.5, etc.). To streamline the categorization process, we decided to associate each numerical value with a specific litter category (microlitter, mesolitter, and macrolitter) based on classifications found in the literature [94,102,103]. In most of the articles analyzed (36 articles), a single category of litter was considered; more than one category of litter was considered in 33 articles; and 18 articles did not report information on the dimensions of the beach litter analyzed. Specifically, 30 articles focused only on the macrolitter category, while six articles focused only on the microlitter category. One article considered both the microlitter and mesolitter categories, while 17 articles considered both the mesolitter and macrolitter categories, and 15 articles considered all categories (microlitter, mesolitter, and macrolitter) (Figure 5). Most studies have focused on the study of macrolitter; this is probably due to the greater ease in investigating this component. An interesting observation concerns the absence, in many studies, of information regarding the specific category of beach litter being investigated. It should be highlighted that the size of the beach litter is an important factor, both in choosing the sampling methodology to apply in the study [103] and in comparing the results between different studies. For this reason, it would always be appropriate and necessary to specify which category is being investigated.

Out of the 87 studies in which the field sampling of beach litter was conducted, only 36 provided precise information about the investigated beach litter. Among these, the majority examined both surface litter and litter buried in the sand (52.78%), while the remaining studies focused solely on surface litter (38.89%) or buried litter (8.33%). In this context, the choice of beach litter type to investigate may depend on various factors. For instance, many studies analyzing microlitter involve sifting the first centimeters of sand in a designated area to better identify small litter particles. Another reason pertains to the type of beach under investigation; it is important to note that, when examining beaches within protected areas, including Sites of Community Importance (SIC sensu EU Habitat Directive 92/43/EEC; [130]), excavation should typically be avoided to prevent damage to the dune system. However, to our knowledge, there are currently no established guidelines in the literature specifying which of these types of litter should be investigated to achieve an optimal assessment.

## 4. Characterization of Beach Litter Abundance and Distribution in Relation to Psammophilous Plant Species and Communities

Only 18 studies out of 103 present at the Mediterranean Basin level concurrently examined psammophilous plant species and communities while investigating the occurrence of beach litter in dune systems (Table 1). The methodologies employed to investigate the relationship between psammophilous plant species and communities and the presence of beach litter exhibited considerable similarity in this context.

**Figure 6:**
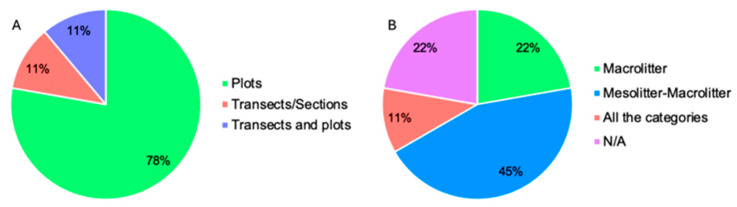
Percentage of articles regarding each methodology used for the study of beach litter in relation to plant species and communities (**A**) and that investigated the different categories of beach litter in relation to plant species and communities (**B**). All categories = microlitter, items up to 5 mm in the longest dimension; mesolitter, items from 5 mm to 25 mm in the longest dimension; and macrolitter, items above 25 mm in the longest dimension [103]; N/A = data not indicated.

Firstly, within the majority of the studies (14 in total), plots were used, although with different dimensions. Two articles [55,131] reported the use of both transects and plots, each employing different dimensions, and two studies [111,120] exclusively used transects/sections (Figure 6A). The main plot size used to investigate the relationship between beach litter and psammophilous plant species and communities was 1 × 1 m, with five studies exclusively adopting this size. Additionally, four studies uniquely used plot sizes of 2 × 2 m, while three studies used dimensions of 25 × 25 cm, and only one study used 10 × 10 m plots (Table 1). Three studies used varied plot sizes [49,50,131]; among these, Mo et al. [50] analyzed plots of three distinct sizes (16 m^2^, 4 m^2^, and 1 m^2^) to identify which of these dimensions was optimal for the investigation of beach litter. This study found that plots of 16 m^2^ and 4 m^2^ allowed for the adequate investigation of the presence and distribution of beach litter, with 16 m^2^ plots appearing to be the most appropriate size [50]. Additionally, there is a wealth of evidence in the literature indicating that a plot size of 4 m^2^ is optimal in estimating the percentage of plant species cover in a dune system, e.g., [66,88,132,133,134]. In light of these data, the use of 4 m^2^ plots would be considered more appropriate in the study of beach litter in relation to the plant species and communities typical of dune systems. However, it is necessary to point out that this unit of measurement could be more suitable for the study of meso- and macrolitters. To allow for the study of the microlitter component as well, within the 4 m^2^ plot, soil samples could be acquired and analyzed—for example, by taking the top few centimeters of substrate and taking care not to damage the plant species present in the surrounding areas.

Regarding the dimensions of the beach litter investigated, in this case, to simplify the presentation of the results, we also assigned the dimensions reported in the articles to a specific category (microlitter, mesolitter, and macrolitter) based on the classifications reported in the literature [90,98,99]. Among the articles analyzed, eight considered both the mesolitter and macrolitter categories, while four focused solely on macrolitter (Figure 6B). Only two articles examined all categories simultaneously (microlitter, mesolitter, and macrolitter), and four articles did not provide this information (Figure 6B). Consequently, it is apparent that the litter sizes primarily investigated in relation to psammophilous plant species and communities are those larger than 5 mm. This preference likely stems from the ease of identifying such litter within the dune system. Moreover, there is a dearth of studies addressing the impact of microlitter on psammophilous plant species and communities.

Secondly, most of the articles were based on data collected during a single sampling (nine articles); in three studies, the sampling of the same points was repeated twice; in one study, the samplings were repeated seasonally; and this information was not reported in five articles (Table 1). As mentioned in the previous paragraph, a single sampling can provide basic information about the presence of beach litter on a specific beach, while a greater number of samplings enables the better investigation of the accumulation and distribution of beach litter [102]. Hence, even in this context, it is not possible to determine the requisite number of samplings to study the relationship between beach litter and psammophilous plant species and communities for a single beach. However, it is important to note that, at the Mediterranean Basin level, there is a lack of information regarding the variation in the distribution and quantity of beach litter throughout the seasons or over an entire year in relation to the presence of psammophilous plant species and communities. Therefore, to obtain greater insight into how dune habitats can impact the beach litter component in the long term, studies are needed in which the sampling of the same sites is repeated more frequently (e.g., seasonally or monthly).

Taking into account the specific focus of each article, the research highlighted how the majority (13 in total) focused on dune habitats and their capacity to trap beach litter [1,49,50,52,53,55,58,59,60,111,120,131,135]. Additionally, three articles examined one or more plant species [54,61,62], while only two addressed dune vegetation at both the habitat and plant species levels [51,136].

**Table 1:** List of the scientific studies at the Mediterranean Basin level that have investigated the relationship between beach litter and psammophilous plant species and communities. The habitat classification follows the EU Habitats Directive (Directive 92/43/EEC; [130]), while the taxonomy of plant species is based on the International Plant Names Index (IPNI) [137]. N/A = data not indicated. (*) = priority habitat in Europe.

Article	Article’s Focus		No. Beach	Sampling Rate	Sampling Unit
Poeta et al., 2014 [1]	Habitat	1210; 2110; 2120; 2210; 2230; 2250 *; 2260; 9340	5	Once	2 m × 2 m
de Francesco et al., 2018 [131]	Habitat	1210; 2110; 2120; 2230; 2250 *; 2260	3	N/A	Different dimensions
Šilc et al., 2018 [58]	Habitat	1210; 2110; 2120; 2190; 2270 *	1	Once	2 m × 2 m
de Francesco et al., 2019 [59]	Habitat	1210; 2110; 2120; 2230; 2250 *; 2260; 2270 *	7	Once	2 m × 2 m
Menicagli et al., 2019 [61]	Plant species	*Elytrigia juncea* (L.) Nevski, *Ammophila arenaria* (L.) Link, and *Glaucium flavum* Crantz	1	N/A	25 cm × 25 cm
Battisti et al., 2020 [135]	Habitat	1210; 2110	1	N/A	10 m × 10 m
Menicagli et al., 2020 [62]	Plant species	*Elytrigia juncea* (L.) Nevski and *Sporobolus pungens* Kunth	1	N/A	25 cm × 25 cm
Cresta and Battisti, 2021 [120]	Habitat	1210; 2110; 1410	1	Twice	90 m
Di Febbraro et al., 2021 [60]	Habitat	The entire psammophilous geosigmetum	6	Once	2 m × 2 m
Gallitelli et al., 2021 [49]	Habitat	2110	1	Once	Different dimensions
Mo et al., 2021 [50]	Habitat	1210; 2110; 2120	3	Twice	16 m^2^; 4 m^2^; 1 m^2^
Battisti et al., 2023 [51]	Habitat and plant species	1210; *Salsola kali* L.	1	Once	1 m × 1 m
Calderisi et al., 2023 [52]	Habitat	1210; 2110; 2120; 2210	1	Twice	1 m × 1 m
Egea et al., 2023 [111]	Habitat	1410	1	Seasonally	100 m × 8 m
Gallitelli et al., 2023 [53]	Habitat	2110; 2120; 1410; 3150	1	Once	1 m × 1 m
Gallitelli et al., 2023 [54]	Plant species	*Echinophora spinosa* L., *Limbarda crithmoides* (L.) Dumort., *Anthemis maritima* L., *Pancratium maritimum* L., *Elytrigia juncea* (L.) Nevski, and *Salsola kali* L.	1	Once	1 m × 1 m
Mancuso et al., 2023 [55]	Habitat	1210; 2120	2	Once	100 m; 1 m × 1 m
Menicagli et al., 2023 [136]	Habitat and plant species	1210; 2110; 2120; *Elytrigia juncea* (L.) Nevski and *Carpobrotus* spp.	1	N/A	25 cm × 25 cm

Considering the coastal dune habitats examined across the literature, with reference to the EU Habitats Directive (Directive 92/43/EEC; [130]), it becomes evident that the most studied habitats include (1) the annual vegetation of drift lines (Habitat 1210), investigated in 12 articles; (2) the embryonic shifting dunes (Habitat 2110), also explored in 12 articles; (3) and the shifting dunes along the shoreline with *Ammophila arenaria* (L.) Link (Habitat 2120), assessed in 10 articles (Table 1). Some particular habitats that emerged from the research are the Mediterranean salt meadows (*Juncetalia maritimi*; Habitat 1410), investigated in three articles [53,111,120]; the humid dune slacks (Habitat 2190), investigated in a single article [58]; and the natural eutrophic lakes with *Magnopotamion*- or *Hydrocharition*-type vegetation (Habitat 3150), investigated in a single article [53]. Compared to the total number of articles addressing dune habitats, six studies encompassed the entire psammophilous geosigmetum [1,52,58,59,60,131]. Five articles examined only some habitats within this geosigmetum [50,53,55,120,135]. Three articles focused on a single dune habitat [49,51,111], while, for one article, it was not possible to ascertain definitively whether the series of habitats on the specific beach investigated was complete [136]. Once again, due to the lack of specific guidance on the optimal method for the assessment of the relationship between beach litter and psammophilous plant species and communities, determining the correct approach at the habitat level remains challenging. However, to gain a clearer understanding of the distribution and quantity of beach litter throughout the entire dune system, this anthropic component should be evaluated within the entire psammophilous geosigmetum.

Taking into consideration articles that examined plant species, Menicagli et al. [61] focused on *Elytrigia juncea* (L.) Nevski, *Ammophila arenaria* (L.) Link, and *Glaucium flavum* Crantz. The aim of their study was to assess the effects of the deposition of two types of bags—a non-biodegradable variety and a compostable alternative—on these plant species, employing a field seed sowing experiment. The findings of their research indicated that both types of bags exhibited a detrimental effect on the sexual recruitment success and the growth of newly established plants [61]. Similar findings were also reported in the study conducted by Menicagli et al. [62], which examined a distinct set of plant species: *E. juncea* and *Sporobolus pungens* Kunth. More recently, the role of specific psammophilous plant species in trapping beach litter has been investigated. In particular, Battisti et al. [51], focusing on the plant species *Salsola kali* L. in relation to mesolitter and macrolitter, observed that plots where this species was present exhibited the higher accumulation of litter, including significantly longer items, compared to the plots without vegetation. Additionally, Gallitelli et al. [54] focused on the capacity of vegetation to capture litter, encompassing all its categories (micro-, meso-, and macro-litter) across multiple species (Table 1) and examining their morphological structures. This study demonstrated that vegetation plays a role in litter entrapment along beaches, with interspecific variations correlating with the plant morphology [54]. Lastly, Menicagli et al. [136], focusing on the plant species *E. juncea* and *Carpobrotus* spp., observed differential effects of buried non-biodegradable plastic bags and biodegradable/compostable plastic bags on native and invasive species. Specifically, both variants of plastic bags attenuated the performance of the native species while concurrently fostering the proliferation of the invasive *Carpobrotus* spp. within dune ecosystems [136].

Another intriguing aspect pertains to the examination of beach litter in habitats invaded by alien species. Three studies emerged from this research that concurrently assessed the presence of invasive alien species and beach litter. Specifically, two studies [49,52] investigated the differences in litter accumulation between habitats invaded by the invasive alien species *Carpobrotus acinaciformis* L. Bolus and habitats where this species was absent, revealing that invaded habitats tend to accumulate a greater number of objects, even with a large surface area, compared to native habitats. Meanwhile, in the study by Menicagli et al. [136], the impact of different types of litter on the performance of the invasive *Carpobrotus* spp. was explored. Studies relating to this topic are still limited, although a new contribution was recently added [56]. It is clear that further studies are needed to better understand the possible interactions between beach litter and habitats invaded by alien species.

Finally, a significant consideration involves assessing how the quantity and distribution of beach litter vary across different dune habitats in response to tourism. This review revealed that only a limited number of studies (three in total) have directly [50] or indirectly [52,55] examined the impact of tourism. Given the prevalence of highly touristic beaches in the Mediterranean Basin, it is noteworthy that there is a lack of detailed studies evaluating the proportion of beach litter deposited in various dune habitats attributable to tourism. However, it should be kept in mind that, on tourist beaches, beach cleaning operations are often carried out, and, in some cases, they are not adequate (such as mechanical beach cleaning), which can lead to errors in the assessment of beach litter in relation to tourism. Nevertheless, further studies are required, which must be designed in a manner that ensures that beach cleaning operations do not interfere with data collection.

## 5. Materials and Methods

To achieve our aims, a search was conducted on Scopus, the Elsevier scientific database (www.scopus.com; accessed on 7 January 2024), by intersecting three groups of keywords to find articles reporting the presence of beach litter in the coastal environment within the Mediterranean Basin, in correlation with psammophilous vegetation, up to December 2023. The first group included the keywords “marine litter”, “beach litter”, and “plastic”; the second group included only one keyword, which was “Mediterranean Basin”; the third group included the keywords “plant” and “habitat”. Each word from the first group was combined simultaneously with the only word present in the second group and with each word from the third group. In total, we obtained six different results by setting the search strings as (1) (ALL (“marine litter”) AND ALL (“mediterranean basin”) AND ALL (“habitat”)); (2) (ALL (“marine litter”) AND ALL (“mediterranean basin”) AND ALL (“plant”)); (3) (ALL (“beach litter”) AND ALL (“mediterranean basin”) AND ALL (“habitat”)); (4) (ALL (“beach litter”) AND ALL (“mediterranean basin”) AND ALL (“plant”)); (5) (ALL (“plastic”) AND ALL (“mediterranean basin”) AND ALL (“habitat”)); (6) (ALL (“plastic”) AND ALL (“mediterranean basin”) AND ALL (“plant”)). Moreover, we limited each search to five subject areas, which were “Environmental Science”, “Agricultural and Biological Sciences”, “Earth and Planetary Sciences”, “Chemistry”, and “Multidisciplinary”.

All obtained articles were reviewed and classified into three different categories: articles concerning coastal ecosystems, articles concerning marine ecosystems, and articles concerning freshwater ecosystems. Subsequently, the scope of the research was narrowed down to encompass the literature specifically addressing sandy coastal ecosystems within the Mediterranean Basin. Specifically, only studies concerning beaches overlooking the Mediterranean Basin were considered. For countries with coastlines along the Atlantic Ocean or the Red Sea, or those including extra-Mediterranean territories (France, Morocco, Spain, Israel, and Egypt), the data were filtered to exclude these situations. In the final phase, all articles were examined thoroughly to determine how many of them considered psammophilous plant species and communities in relation to the presence of beach litter. Specifically, each article concerning this topic underwent detailed analysis to identify the plant species examined.

The classification outlined in the EU Habitats Directive (Directive 92/43/EEC; [130]) was referenced to identify all considered habitats; in the papers where it was not specified, and for non-UE countries, the habitats were identified based on the site descriptions and the structural species reported.

The taxonomical treatment of all plant species investigated has been revised using the International Plant Names Index (IPNI) [137] as a reference.

## 6. Challenges and Future Directions

**Figure 7:**
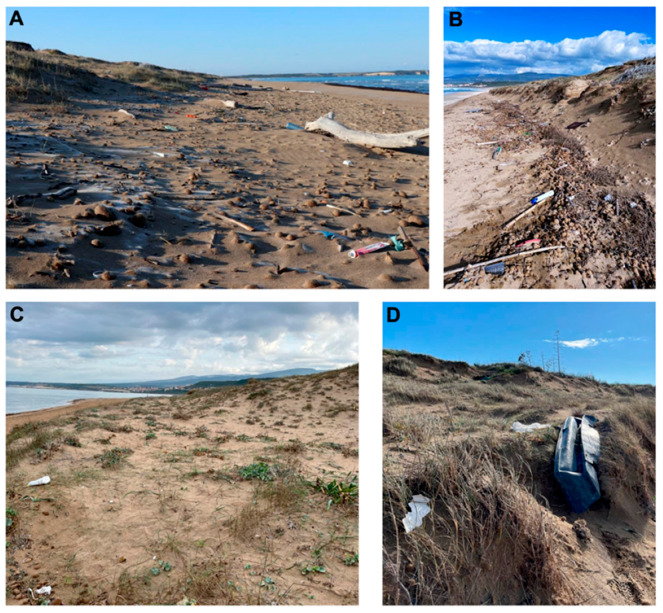
Examples of beach litter found in dune systems, both in the unvegetated sections of the dunes (**A**,**B**) and in vegetated areas (**C**,**D**) (photos by Giulia Calderisi).

Marine beach litter poses significant hazards to ecosystems, plants, and animals (including humans). It can cause significant detrimental effects, such as entanglement, internal injuries, and toxic stress in marine animals, including whales, turtles, birds, and fish [13]. Microplastics, prevalent in oceans, are especially harmful, leading to health issues in organisms, including infertility, growth inhibition, internal or external injuries, and the obstruction of bodily passages, among others [33,34,35]. It has been demonstrated that, through the food chain, they also affect human health [36,37,38]. Glass, metal, cigarette butts, condoms, ceramics, and nails are common types of litter found on beaches. These items can harm both wildlife and humans, and they have been classified as hazardous anthropogenic litter [138,139]. Ultimately, the accumulation of beach litter is a relevant current environmental problem since it can impact all aspects of biodiversity and ecosystem services, affecting biological populations, communities, and biogeochemical cycles across various ecological domains [83,84,85] (Figure 7).

Our study enabled us to construct an overview of research concerning beach litter in the Mediterranean Basin, specifically focusing on the correlation with psammophilous plant species and communities. Several issues arise from the literature when considering both the topic of beach litter in general and its relation to the plant species and communities typical of dune systems.

First of all, despite the fact that the number of studies investigating beach litter in coastal dune systems has significantly increased in recent years, this topic remains relatively underexplored, and the current information is concentrated in a few countries, especially when compared to research on litter in marine ecosystems. This gap could be due to the difficulty in drawing on the data present in the grey literature or in the technical reports prepared by local institutions, which are generally not covered by large scientific databases. Specifically, a significant lack of data can be highlighted in the southern part of the Mediterranean Basin, which constitutes a substantial portion of the coast. The scarcity of studies on the interplay between beach litter and coastal environments (particularly plant species and communities) in these countries may stem from various factors, such as limited economic resources, low awareness of their interconnectedness, and the prioritization of research in domains that are perceived to be more important. In addition, political instability and security concerns in several areas may further hinder data collection and research efforts in these regions. In any case, this lack of data, which also affects other parts of the Mediterranean Basin, prevents us from having a homogeneous information framework at the basin level, limiting the ability to draw comprehensive conclusions. Moreover, despite the availability of numerous technical manuals, e.g., [5,101,102,125], providing guidelines for the study of beach litter, there is a tendency to follow specific protocols, with the result of collecting information that is difficult to compare, often stemming from an expert-based approach. The variability in the applied methodologies could lead to inconsistent findings, making it challenging to draw universally applicable conclusions and complicating the process of comparing data across different beaches and countries. For this reason, it is necessary to develop a general strategy, common guidelines, and an operative manual that is effective at a biogeographical scale for the Mediterranean Basin, highlighting what has been discovered so far and outlining standardized techniques for beach litter analysis that can be replicated across different beaches and countries. This protocol should establish specific measurement units appropriate for comprehensive beach analysis and establish a standardized classification system for beach litter. Employing uniform measurement units and litter categorization would enhance the data’s precision and comparability. Additionally, the protocol should include the monitoring of the beach through repeated sampling over time and the analysis of consistent sampling points to assess the accumulation and distribution of beach litter.

Upon closer examination of the correlation between beach litter and psammophilous plant species and communities, a significant dearth of data at the Mediterranean Basin level becomes evident. Furthermore, despite the methodologies employed in this specific context exhibiting greater uniformity, notable discrepancies persist among different research endeavors. Primarily, the majority of these investigations rely on data gathered mainly from singular or two-times samplings of the beaches during a year, revealing a lack of studies examining the temporal variability in the anthropic beach litter distribution in relation to dune habitats over extended periods. Furthermore, although the majority of the studies utilize plots as a methodology to explore the relationship between beach litter and plant species and communities, the choice of plot size varies across studies. In addition, a relatively limited number of studies have assessed the presence of beach litter in relation to the entire psammophilous geosigmetum, with the majority concentrating on specific plant species or delving into one or two dune habitats. These points underscore the requirement for further research to enhance our understanding of the impact of beach litter on sandy dune systems and, particularly, on psammophilous plant species and communities. In this sense, the need to use a standardized protocol, applicable across various beaches and even within different countries, is clear. This protocol should consider several factors. Primarily, to assess the presence and distribution of beach litter throughout the entire dune system, it would be necessary to use broad monitoring procedures that include monthly surveys over the course of a year or, at least, throughout the four seasons. These surveys should be consistently conducted at the same points to ensure the repeatability and accuracy of the survey. Furthermore, in the study of beach litter, particularly meso- and macrolitter, plots equal in size should be used; for example, plots of 4 m^2^ could be used, as discussed in the previous paragraph. In fact, this unit allows a comprehensive analysis of beach litter and, at the same time, an exhaustive analysis of psammophilous plant species and communities. Additionally, to understand the variability in the beach litter distribution across the various habitats within dune systems, it is advisable to position these plots along a transect spanning the entire psammophilous geosigmetum. In order to collect data that can be compared at the levels of different beaches and different countries, the analyzed habitats should also be standardized—for example, using the habitats of the EU Habitats Directive (Directive 92/43/EEC; [130]) as a general reference. This approach would expand the scope of habitats under consideration, providing more comprehensive insights into this matter. It is crucial to highlight that, for the sake of ensuring data comparability among various countries, taxonomic uniformity for the flora and consistency in the manual employed for dune habitat identification are required. These endeavors could hold paramount importance in shaping innovative management and conservation strategies for these inherently fragile ecosystems affected by the beach litter problem.

Similarly, it would be necessary to increase the number of plant species analyzed in relation to the presence of beach litter, focusing not only on endangered or native plants but also considering alien plant species that invade dune systems in different countries. These studies should consider two extremely important factors. Firstly, they should assess the impact of beach litter, in all its forms, on the early stages of the life cycles of structural psammophilous plant species typical of dune systems. Currently, to our knowledge, in the Mediterranean Basin, there are only a few studies that have evaluated this particular effect, focusing solely on certain categories of litter and certain psammophilous plant species [61,62,136]. Expanding the range of plant species and beach litter categories analyzed would lead to the greater comprehension of how this anthropic component affects the early stages of plant species’ development and growth. This enhanced understanding would enable the assessment of such information in the formulation of management and conservation strategies for dune systems.

Lastly, given the significant economic role of tourism in the Mediterranean Basin, it would be necessary to also take this variable into account in the study and management of beach litter. To achieve this, it is necessary to expand the scope of research to evaluate how tourism affects the accumulation of beach litter in dune systems. This involves implementing more frequent monitoring through repeated sampling, such as biannual assessments conducted before and after the tourist season. These samplings should be scheduled either at the same time or before beach cleaning campaigns to ensure the integrity of the data collected. Once again, this information could be used at a regional level to enhance the management and conservation strategies for these fragile ecosystems.

## 7. Conclusions

This study presents a critical review of the literature on beach litter in the Mediterranean Basin, with a focus on its relationship with psammophilous plant species and communities.

Research on the effects of beach litter on psammophilous plant communities in the Mediterranean Basin reveals significant gaps, particularly in the Basin’s southern countries, which could be attributed to a variety of factors, including limited research resources, low awareness, and political and security instability. The current attempts are limited by discrepancies in the protocols used for data collection, which make it difficult to compare the results across beaches and countries and draw generalizable conclusions. As a result, there is a clear need for a standardized approach that gives guidance for the monitoring of beach litter throughout the Mediterranean Basin, as well as in relation to psammophilous plant species and communities. In this regard, research should be broadened to encompass a broader array of plant species, both native and invasive, as well as all dune habitats, to thoroughly evaluate the ecological effects of beach litter on these fragile ecosystems. Standardizing data collection and extending the extent of research are essential phases in the development of sustainable management procedures and more solid conservation strategies for the Mediterranean coastal ecosystems.

## Data Availability

Detailed data are available from the corresponding author upon request.

## Supplementary Materials

The following supporting information can be downloaded at: https://www.mdpi.com/article/10.3390/plants13223125/s1, Table S1: Publications relating to the topic of beach litter present in coastal environments. The articles in which psammophilous plant species and communities were considered are highlighted in bold. References [140,141,142,143,144,145,146,147,148,149,150,151,152,153,154,155,156,157,158,159,160,161,162,163,164,165,166,167,168,169,170,171,172,173,174,175,176,177,178,179,180,181,182,183,184,185,186,187,188,189,190,191,192,193,194,195,196,197,198,199,200,201] are included in the supplementary materials.
