# Supplementary material for: Unravelling the Nexus of Beach Litter and Plant Species and Communities Along the Mediterranean Coasts: A Critical Literature Review
Source: Plants (Basel). 2024 Nov 6;13(22):3125. doi: 10.3390/plants13223125 (PMC11597917; doi:10.3390/plants13223125)
Supplement: Supplementary file 1 [file plants-13-03125-s001.zip › plants-3272546-supplementary.pdf]

plants

Review

Unravelling the Nexus of Beach Litter and Plant Species and Communities Along the Mediterranean Coasts: A Critical Literature Review

Giulia Calderisi, Donatella Cogoni and Giuseppe Fenu *

Department of Life and Environmental Sciences, University of Cagliari, Viale Sant'Ignazio da Laconi 13, 09123 Cagliari, Italy; giulia.calderisi@unica.it (G.C.); d.cogoni@unica.it (D.C.)
* Correspondence: gfenu@unica.it

Table S1. Publications relating to the topic of beach litter present in coastal environments. The articles in which psammophilous plant species and communities were considered are highlighted in green.

Articles	No. Beach	Location	Sampling method	Sampling units	Sampling rate	Sampling period	Beach litter collection	Beach litter categorization	Beach litter size
Gabrielides et al., 1991 [115]	13 (12 in the Mediterranean Basin)	Cyprus, Israel, Italy, Turkey	Transects/Sections	Lenght: from the waterline to the back of the beach. The transect width varied.	Multiple	Between mid 1988 and mid 1989	yes	yes	> 1 cm
Shiber and Barrales-Rienda, 1991 [121]	5	Lebanon	Transects/Sections	Sections with an area of 150 m^2 or 300 m^2	Once for four beach. In AUB beach three times	March and April 1988	yes	yes	≥ 0.2 cm
Golik and Gertner, 1992 [116]	6	Israel	Transects/Sections	Lenght: from the waterline to the back of the beach. 5-m-wide.	Monthly	Between May 1988 and May 1989	no	yes	> 2 cm
Bowman et al., 1998 [112]	6	Israel, Gaza strip	Transects/Sections	Lenght: 50 m	Multiple	Between July 1990 and October 1991	no	yes	N/A
Tudor et al., 2002 [105]	33 (7 in the Mediterranean Basin)	Malta, Spain, Tunisia, Turkey	Transects/Sections	Lenght: 100 m	Multiple	N/A	N/A	yes	N/A
Martínez Ribes et al., 2007 [117]	32	Spain	Transects/Sections	Lenght: from the waterline to the back of the beach. 2-m-wide	Once in Minorca and Ibiza. Monthly in Mallorca	2005	yes	yes	> 0.1 cm

Kordella et al., 2013 [140]	78	Greece	Beach clean-ups	N/A	Once	2006 and 2007	yes	yes	> 1 cm
Laglbauer et al., 2014 [113]	6	Slovenia	Transects and plots	Lenght: 50 m parallel to the shoreline (from the shoreline to the upper beach limit) Plot: 25 cm^2	Once	July 2012	yes	yes	≥ 2 cm > 0.025 cm and < 0.1 cm
**Poeta et al., 2014 [1]**	5	Italy	Plots	2 m × 2 m	Once	April and May 2012	no	yes	N/A
Katsanevakis, 2015 [106]	2	Greece	Transects/Sections	Lenght: 100 m (from the tide line to the back border of the beach)	N/A	N/A	yes	yes	N/A
Aydın et al., 2016 [107]	13	Turkey	Transects/Sections	100 m (from the shoreline to the back beach)	Once	April 2014	yes	yes	> 2.5 cm
Battisti et al., 2016 [122]	1	Italy	Transects/Sections	A total of 3000 square metres	N/A	N/A	yes	yes	> 0.5 cm
Gönülal et al., 2016 [141]	12	Turkey	N/A	N/A	Multiple	Between October 2013 and April 2015	yes	yes	N/A
Munari et al., 2016 [114]	5	Italy	Transects/Sections	50 m parallel to the shoreline (from the water edge to the back of the beach)	N/A	From May to June 2015	yes	yes	> 2 cm
Poeta et al., 2016 [126]	1	Italy	Transects/Sections	100 m (between the sea line and the back dune's woody vegetation)	Seasonally	Between spring 2014 and winter 2015	yes	yes	≥ 2.5 cm

Poeta et al., 2016 [108]	5	Italy	Plots	2 m × 2 m	Once	Between April and May 2012	no	yes	N/A
Alshawafi et al., 2017 [142]	1	Morocco	Transects and plots	Lenght: from the low tide to the end of the shoreline<br>Plots: 50 cm × 50 cm	Seasonally	2015	yes	yes	> 0.125 cm
Lots et al., 2017 [143]	23 (10 in the Mediterranean Basin)	Bosnia, France, Greece, Israel, Italy, Spain, Turkey	Transects/Sections	40 m (at the high tide line)	Multiple	Between June 2015 and January 2017	yes	yes	< 0.5 cm
Munari et al., 2017 [144]	5	Italy	Plots	50 cm × 50 cm	N/A	May 2015	yes	yes	≧ 0.5 cm
Pasternak et al., 2017 [145]	8	Israel	Transects/Sections	Lenght: 100 m<br>Width: from the waterline to a physical border on the backshore	Multiple	From June 2012 to March 2015	no	yes	> 2.5 cm
Portman and Brennan, 2017 [146]	1	Israel	Transects/Sections	N/A	Multiple	From April to July 2016	N/A	yes	≥ 2 cm
Zhang, 2017 [147]									< 0.5 cm
**de Francesco et al., 2018 [131]**	3	Italy	Transects and plots	Plots of about 200 m² for sampling small–medium litter<br>Transects of 1 km for sampling large litter	N/A	In spring (2014–2015)	yes	yes	≥ 2 cm
Duncan et al., 2018 [148]	17	Cyprus	Transects and plots	N/A	N/A	Between July and August 2016	yes	yes	> 0.1 cm and < 0.5 cm

Giovacchini et al., 2018 [109]	11	Italy	Transects/Sections	100 m (from the shoreline to the beginning of the psammophilous vegetation)	Multiple	From January 2014 and December 2015	yes	yes	> 2.5 cm
Karkanorachaki et al., 2018 [149]	4	Greece	Transects and plots	40 × 40 cm	Multiple	From November 2013 to March 2015	yes	yes	> 0.2 cm
Loizidou et al., 2018 [150]	9	Cyprus	Beach clean-ups	A total area of 20,980 m²	Twice	Between May and August (2016 and 2017)	yes	yes	N/A
Maziane et al., 2018 [123]	14 (12 in the Mediterranean Basin)	Morocco	Transects/Sections	Lenght: from the strandline to the beach landward boundary. 100-m-wide	N/A	From November to December 2015	yes	yes	> 2.5 cm
Prevenios et al., 2018 [118]	4	Greece	Transects/Sections	Lenght: from the water line to the back of the beach. 100-m-wide	Multiple	From July 2014 to October 2015	yes	yes	≥ 2.5 cm
**Šilc et al., 2018 [58]**	1	Montenegro	Plots	2 m × 2 m	Once	May 2017	yes	yes	N/A
Vlachogianni et al., 2018 [151]	31	Albania, Bosnia and Herzegovina, Croatia, Greece, Italy, Montenegro, Slovenia	Transects/Sections	100 m × 10 m	Multiple	From October 2014 to April 2016	yes	yes	> 2.5 cm
Asensio-Montesinos et al., 2019 [152]	56	Spain	Transects/Sections	100 m	Twice	March and August 2018	N/A	yes	N/A

Asensio-Montesinos et al., 2019 [153]	56	Spain	Transects/Sections	100 m	N/A	From March to April 2018	N/A	yes	N/A
Battisti et al., 2019 [154]	1	Italy	Transects/Sections	15,000 square metres	N/A	March 2018	yes	yes	N/A
Constant et al., 2019 [155]	2	France	Plots	0.5 × 0.5 m	Twice	February and March 2016	yes	yes	< 0.8 cm
**de Francesco et al., 2019 [59]**	7	Italy	Plots	2 m × 2 m	Once	April and May 2018	yes	yes	> 2.5 cm
Gündoğdu and Çevik, 2019 [127]	13	Turkey	Transects and plots	1 m × 1 m	N/A	May 2018	yes	yes	> 0.5 cm
**Menicagli et al., 2019 [61]**	1	Italy	Plots	25 cm × 25 cm	N/A	February 2018	no	no	N/A
Mokos et al., 2019 [156]	3	Croatia	Transects/Sections	100 m or 50 m	Once	Between March and May 2018	yes	yes	> 2.5 cm
Nachite et al., 2019 [157]	14 (12 in the Mediterranean Basin)	Morocco	Transects/Sections	100 m	Multiple	Autumn (2015 and 2016) and spring (2016 and 2017)	yes	yes	> 2.5 cm
**Battisti et al., 2020 [135]**	1	Italy	Plots	10 x 10	N/A	N/A	yes	no	≳ 2.5 cm
Gjyli et al., 2020 [12]	5	Albania	Transects/Sections	Lenght: between the strandline and all the way towards the back of the beach. 100-m-wide	Once	April 2018	yes	yes	> 2.5 cm

Grelaud and Ziveri, 2020 [124]	24	Croatia, Cyprus, Greece, Italy, Malta, Spain	Transects/Sections	Lenght: from the water line to the back of the beach. 100-m-wide	Multiple	2017 and 2019	yes	yes	≥ 0.1 cm
Lechthaler et al., 2020 [158]									≥ 2.5 cm
**Menicagli et al., 2020 [62]**	1	Italy	Plots	25 cm × 25 cm	N/A	From 2018 to 2019	no	no	> 2.5 cm
Mghili et al., 2020 [110]	5 (3 in the Mediterranean Basin)	Morocco	Transects/Sections	100 m × 20 m	Seasonally	2019	yes	yes	> 2.5 cm
Mokos et al., 2020 [159]	1	Croatia	Transects/Sections	100 m × 10 m	Seasonally	June-October 2018 and January-April 2019	yes	yes	> 2.5 cm
Papachristopoulou et al., 2020 [160]	4	Greece	Beach clean-ups	Full beach	Seasonally	From August 2017 to August 2018	yes	yes	≥ 2.5 cm
Vlachogianni et al., 2020 [161]	23	Croatia, Cyprus, France, Greece, Italy	Transects/Sections	100-metre stretch from the strandline to the back of the beach	Twice	From mid-September to mid-October and from mid-November to mid-December 2018	yes	yes	> 2.5 cm

Asensio-Montesinos et al., 2021 [162]	12	Spain	Transects/Sections	Lenght: from the landward beach limit to the water line. 100-m-wide	Multiple	February, March, and April 2019	yes	yes	> 2.5 cm
Asensio-Montesinos et al., 2021 [163]	5	Spain	Transects/Sections	Full beach, from the strandline to the backshore area	Multiple	From December 2020 to March 2021	no	yes	N/A
Benaissa et al., 2021 [164]	10	Algeria	Transects/Sections	Between the water line and the beach backshore over a length of 100 m	Once	Between March and April 2019	no	yes	> 5 cm
Bozzeda et al., 2021 [165]	1	Italy	Transects and plots	From the shoreline to the base of the dune; 30 cm × 30 cm	N/A	April 2019	yes	yes	> 0.2 cm
Camedda et al., 2021 [166]	11	Italy	Transects/Sections	100 m, 33.3 m, and 10 m	Seasonally	Spring 2013; autumn 2013; spring 2014; autumn 2015; spring 2016; autumn 2016	yes	yes	> 0.1 cm
Cesarini et al., 2021 [119]	1	Italy	Transects and plots	80 m; 20 cm × 20 cm	Once	November 2019	yes	yes	> 0.25 cm
**Cresta and Battisti, 2021 [120]**	1	Italy	Transects/Sections	90 m	Twice	April and May	yes	yes	≥ 1 cm
**Di Febbraro et al., 2021 [60]**	6	Italy	Plots	2 m × 2 m	Once	April–May 2018	yes	yes	> 2.5 cm
Expósito et al., 2021 [167]	6	Spain	Plots	40 cm × 40 cm	N/A	September-October 2018	yes	yes	< 0.4 cm

Fanini and Guittard, 2021 [168]	2	Greece	Transects/Sections	400 m; delimited on the long-shore dimension by groynes, and with the beach width defined by the seaside road presence	Multiple	From April 2019 to December 2019	yes	yes	> 2.5 cm
Fortibuoni et al., 2021 [169]	64	Italy	Transects/Sections	33 m stretch along the strandline to the first barrier at the back of the shoreline section	Seasonally	From 2015 to 2018	yes	yes	> 2.5 cm
Frigione et al., 2021 [170]	1	Italy	Transects and plots	From the strandline to the base of dunes; 30 cm × 30 cm	Once	April 2018	yes	yes	≷ 0.5 cm
**Gallitelli et al., 2021 [49]**	1	Italy	Plots	Different dimensions	Once	N/A	yes	yes	> 0.5 cm
Mandić et al., 2021 [171]	2	Montenegro	Transects/Sections	Lenght: from the shoreline up to the hinterland of the beach. 100-m-wide	Seasonally	Autumn 2018, winter 2018, and spring 2019	yes	yes	> 2.5 cm
Martín-Lara et al., 2021 [172]		Spain							< 0.5 cm
Merlino et al., 2021 [173]	1	Italy	Drone and in situ visual census	3 × 3 m square grid	N/A	N/A	no	yes	> 5 cm
**Mo et al., 2021 [50]**	3	Italy	Plots	16 m^2, 4 m^2, and 1 m^2	Twice	Spring and autumn 2018	no	yes	N/A

Özden et al., 2021 [174]	8	Cyprus	Transects/Sections	5 m × 50 m	Monthly	Between January 2017 and January 2019	yes	yes	≥ 2.5 cm
Phuong et al., 2021 [175]									< 0.5 cm
Romiti et al., 2021 [176]	2	Italy	Transects/Sections	A total of 4.8 ha	Monthly	From April to June 2019	N/A	yes	N/A
Schmid et al., 2021 [177]									N/A
Schmid et al., 2021 [178]									< 0.5 cm
Taïbi et al., 2021 [179]	9	Algeria	Transects and plots	0.5 m × 0.5 m (on the highest waterline)	Multiple	From February to July 2017	yes	yes	≥ 0.1 cm
Bonanno, 2022 [180]									N/A
Cesarini et al., 2022 [181]	1	Italy	Transects/Sections	From the intertidal zone to before the dunes	Monthly	From March to September 2021	yes	yes	> 0.5 cm
Corbau et al., 2022 [182]	5	Italy	Transects/Sections	5-metre-wide	Seasonally	December 2017 and March–July–October 2019	yes	yes	≥ 2.5 cm and < 50 cm
Menicagli et al., 2022 [183]	1	Italy	Transects/Sections	100 m × 20 m	Seasonally	From September 2020 to May 2021	yes	yes	> 2.5 cm
Nithin et al., 2022 [184]									< 0.5 cm

Novillo-Sanjuan et al., 2022 [185]	3	Spain	Plots	50 cm × 50 cm	Twice	July 2018 and November 2018	yes	yes	< 0.5 cm
Orthodoxou et al., 2022 [186]	20	Cyprus	Transects/Sections	100 m in length and the entire width of the monitoring sites	Multiple	January, March, June, and September 2021	yes	yes	> 2.5 cm
Perumal and Muthuramalingam, 2022 [187]									< 0.5 cm
Poeta et al., 2022 [188]	8	Italy	Plots	4 m × 4 m	Monthly	From January 2019 to January 2020	yes	yes	N/A
Prabhu et al., 2022 [189]									< 0.5 cm
Rubin et al., 2022 [190]	6	Israel	Transects/Sections	0.3 m^2	Multiple	From May to September 2021	yes	yes	≥ 0.1 cm
Simon-Sánchez et al., 2022 [191]									< 0.5 cm
Strafella et al., 2022 [192]									< 0.5 cm
Battisti et al., 2023 [21]	1	Italy	Transects/Sections	A total of 14,640 square metres	Monthly	Between December 2020 and February 2021	yes	yes	> 0.5 cm
**Battisti et al., 2023 [51]**	1	Italy	Plots	1 m × 1 m	Once	Summer	yes	yes	> 0.5 cm

**Calderisi et al., 2023 [52]**	1	Italy	Plots	1 m × 1 m	Twice	May and October 2022	no	yes	> 0.5 cm
Celine et al., 2023 [193]	N/A	Lebanon	Transects and plots	N/A	N/A	November 2019	yes	yes	< 0.5 cm
Cesarano et al., 2023 [14]									≥ 0.5 cm
**Egea et al., 2023 [111]**	1	Spain	Transects/Sections	100 m x 8 m	Seasonally	N/A	yes	Yes	> 0.5 mm
Fagiano et al., 2023 [194]	6	Spain	Plots	0.25 m × 0.25 m	Multiple	2019	yes	yes	< 0.5 cm
**Gallitelli et al., 2023 [53]**	1	Italy	Plots	1 m × 1 m	Once	N/A	yes	yes	> 0.5 cm
**Gallitelli et al., 2023 [54]**	1	Italy	Plots	1 square metre	Once	August 2021	yes	yes	> 0.3 cm
John et al., 2023 [195]									< 0.5 cm
Ma et al., 2023 [196]									< 0.5 cm
Malli et al., 2023 [197]									< 0.5 cm
**Mancuso et al., 2023 [55]**	2	Italy	Transects and plots	100 m; 1 m × 1 m	Once	May 2022	yes	yes	> 0.5 cm
**Menicagli et al., 2023 [136]**	1	Italy	Plots	25 cm × 25 cm	N/A	From December 2017 to May 2019	N/A	N/A	> 2.5 cm

Mghili et al., 2023 [198]	11 (9 in the Mediterranean Basin)	Morocco	Transects/Sections	100 m × 20 m	Multiple	Between 2018 and 2023 (samplings was not conducted in 2020)	yes	yes	≥ 2.5 cm
Noureddine et al., 2023 [199]	50 (36 in the Mediterranean Basin)	Morocco	EA/NALG technique	N/A	N/A	N/A	N/A	yes	N/A
Rios-Fuster et al., 2023 [200]	N/A	Spain	Coastline observation	N/A	Monthly	From February to July 2021	no	yes	N/A
Vlachogianni and Scoullos, 2023 [201]	9	Greece	Transects/Sections	100-m stretch from the strandline to the back of the beach	Twice	January 2023 and April 2023	yes	yes	> 2.5 cm

References

1. Poeta, G.; Battisti, C.; Acosta, A.T.R. Marine litter in Mediterranean sandy littorals: Spatial distribution patterns along central Italy coastal dunes. *Mar. Pollut. Bull.* **2014**, *89*, 168–173. https://doi.org/10.1016/j.marpolbul.2014.10.011.

12. Gjyli, L.; Vlachogianni, T.; Kolitari, J.; Matta, G.; Metalla, O.; Gjyli, S. Marine litter on the Albanian coastline: Baseline information for improved management. *Ocean. Coast. Manag.* **2020**, *187*, 105108. https://doi.org/10.1016/j.ocecoaman.2020.105108.

14. Cesarano, C.; Aulicino, G.; Cerrano, C.; Ponti, M.; Puce, S. Marine beach litter monitoring strategies along Mediterranean coasts. A methodological review. *Mar. Pollut. Bull.* **2023**, *186*, 114401. https://doi.org/10.1016/j.marpolbul.2022.114401.

21. Battisti, C.; Gallitelli, L.; Vanadia, S.; Scalici, M. General macro-litter as a proxy for fishing lines, hooks and nets entrapping beach-nesting birds: Implications for clean-ups. *Mar. Pollut. Bull.* **2023**, *186*, 114502. https://doi.org/10.1016/j.marpolbul.2022.114502.

49. Gallitelli, L.; Battisti, C.; Olivieri, Z.; Marandola, C.; Acosta, A.T.R.; Scalici, M. Carpobrotus spp. patches as trap for litter: Evidence from a Mediterranean beach. *Mar. Pollut. Bull.* **2021**, *173*, 113029. https://doi.org/10.1016/j.marpolbul.2021.113029.

50. Mo, A.; D'Antraccoli, M.; Bedini, G.; Ciccarelli, D. The role of plants in the face of marine litter invasion: A case study in an Italian protected area. *Mar. Pollut. Bull.* **2021**, *169*, 112544. https://doi.org/10.1016/j.marpolbul.2021.112544.

51. Battisti, C.; Fanelli, G.; Gallitelli, L.; Scalici, M. Dunal plants as sink for anthropogenic marine litter: The entrapping role of Salsola kali L. (1753) in a Mediterranean remote beach (Sardinia, Italy). *Mar. Pollut. Bull.* **2023**, *192*, 115033 https://doi.org/10.1016/j.marpolbul.2023.115033.

52. Calderisi, G.; Cogoni, D.; Loni, A.; Fenu, G. Difference between invasive alien and native vegetation in trapping beach litter: A focus on a typical sandy beach of W-Mediterranean Basin. *Mar. Pollut. Bull.* **2023**, *192*, 115065. https://doi.org/10.1016/j.marpolbul.2023.115065.

53. Gallitelli, L.; Battisti, C.; Scalici, M. Dunal plants intercepting macrolitter: Implications for beach clean-ups. *Mar. Pollut. Bull.* **2023**, *187*, 114585 https://doi.org/10.1016/j.marpolbul.2023.114585.

54. Gallitelli, L.; D'Agostino, M.; Battisti, C.; Cózar, A.; Scalici, M. Dune plants as a sink for beach litter: The species-specific role and edge effect on litter entrapment by plants. *Sci. Total Environ.* **2023**, *904*, 166756. https://doi.org/10.1016/j.scitotenv.2023.166756.

55. Mancuso, M.; Genovese, G.; Porcino, N.; Natale, S.; Crisafulli, A.; Spagnuolo, D.; Catalfamo, M.; Morabito, M.; Bottari, T. Psammophytes as traps for beach litter in the Strait of Messina (Mediterranean Sea). *Reg. Stud. Mar. Sci.* **2023**, *65*, 103057. https://doi.org/10.1016/j.rsma.2023.103057.

58. Šilc, U.; Küzmič, F.; Caković, D.; Stešević, D. Beach litter along various sand dune habitats in the southern Adriatic (E Mediterranean). *Mar. Pollut. Bull.* **2018**, *128*, 353–360. https://doi.org/10.1016/j.marpolbul.2018.01.045.

59. de Francesco, M.C.; Carranza, M.L.; Varricchione, M.; Tozzi, F.P.; Stanisci, A. Natural Protected Areas as Special Sentinels of Littering on Coastal Dune Vegetation. *Sustainability* **2019**, *11*, 5446. https://doi.org/10.3390/su11195446.

60. Di Febbraro, M.; Frate, L.; de Francesco, M.C.; Stanisci, A.; Tozzi, F.P.; Varricchione, M.; Carranza, M.L. Modelling Beach Litter Accumulation on Mediterranean Coastal Landscapes: An Integrative Framework Using Species Distribution Models. *Land* **2021**, *10*, 54. https://doi.org/10.3390/land10010054.

61. Menicagli, V.; Balestri, E.; Vallerini, F.; Castelli, A.; Lardicci, C. Adverse effects of non-biodegradable and compostable plastic bags on the establishment of coastal dune vegetation: First experimental evidences. *Environ. Pollut.* **2019**, *252*, 188–195. https://doi.org/10.1016/j.envpol.2019.05.108.

62. Menicagli, V.; Balestri, E.; Vallerini, F.; Castelli, A.; Lardicci, C. Combined effect of plastic litter and increased atmospheric nitrogen deposition on vegetative propagules of dune plants: A further threat to coastal ecosystems. *Environ. Pollut.* **2020**, *266*, 115281. https://doi.org/10.1016/j.envpol.2020.115281.
105. Tudor, D.T.; Williams, A.T.; Randerson, P.; Ergin, A.; Earll, R.E. The use of multivariate statistical techniques to establish beach debris pollution sources. *J. Coast. Res.* **2002**, *36*, 716–725. https://doi.org/10.2112/1551-5036-36.sp1.716.
106. Katsanevakis, S. Illegal immigration in the eastern Aegean Sea: A new source of marine litter. Mediterr. *Mar. Sci.* **2015**, *16*, 605–608. https://doi.org/10.12681/mms.1463.
107. Aydın, C.; Guven, O.; SALİHOĞLU, B.; KIDEYŞ, A. The Influence of Land Use on Coastal Litter: An Approach to Identify Abundance and Sources in the Coastal Area of Cilician Basin, Turkey. *Turk. J. Fish. Aquat. Sci.* **2016**, *16*, 29–39. https://doi.org/10.4194/1303-2712-v16_1_04.
108. Poeta, G.; Conti, L.; Malavasi, M.; Battisti, C.; Acosta, A.T.R. Beach litter occurrence in sandy littorals: The potential role of urban areas, rivers and beach users in Central Italy. *Estuar. Coast. Shelf Sci.* **2016**, *181*, 231–237. https://doi.org/10.1016/j.ecss.2016.08.041.
109. Giovacchini, A.; Merlino, S.; Locritani, M.; Stroobant, M. Spatial distribution of marine litter along Italian coastal areas in the Pelagos sanctuary (Ligurian Sea—NW Mediterranean Sea): A focus on natural and urban beaches. *Mar. Pollut. Bull.* **2018**, *130*, 140–152. https://doi.org/10.1016/j.marpolbul.2018.02.042.
110. Mghili, B.; Analla, M.; Aksissou, M.; Aissa, C. Marine debris in Moroccan Mediterranean beaches: An assessment of their abundance, composition and sources. *Mar. Pollut. Bull.* **2020**, *160*, 111692. https://doi.org/10.1016/j.marpolbul.2020.111692.
111. Egea, L.G.; Cavijoli-Bosch, J.; Casal-Porras, I.; Yamuza-Magdaleno, A.; Brun, F.G.; Jiménez-Ramos, R. Comparison of macroplastics dynamic across a tidal-dominated coastal habitat seascape including seagrasses, salt marshes, rocky bottoms and soft sediments. *Mar. Pollut. Bull.* **2023**, *196*, 115590. https://doi.org/10.1016/j.marpolbul.2023.115590.
112. Bowman, D.; Manor-Samsonov, N.; Golik, A. Dynamics of litter pollution on Israeli Mediterranean beaches: A budgetary, litter flux approach. *J. Coast. Res.* **1998**, *14*, 418–432.
113. Laglbauer, B.J.; Franco-Santos, R.M.; Andreu-Cazenave, M.; Brunelli, L.; Papadatou, M.; Palatinus, A.; Grego, M.; Deprez, T. Macrodebris and microplastics from beaches in Slovenia. *Mar. Pollut. Bull.* **2014**, *89*, 356–366. https://doi.org/10.1016/j.marpolbul.2014.09.036
114. Munari, C.; Corbau, C.; Simeoni, U.; Mistri, M. Marine litter on Mediterranean shores: Analysis of composition, spatial distribution and sources in north-western Adriatic beaches. *Waste Manag.* **2016**, *49*, 483–490. https://doi.org/10.1016/j.wasman.2015.12.010.
115. Gabrielides, G.P.; Golik, A.; Marino, M.G.; Bingel, F.; Torregrossa, M.V. Man-made garbage pollution on the Mediterranean coastline. *Mar. Pollut. Bull.* **1991**, *23*, 437–441. https://doi.org/10.1016/0025-326X(91)90713-3.
116. Golik, A.; Gertner, Y. Litter on the Israeli coastline. *Mar. Environ. Res.* **1992**, *33*, 1–15. https://doi.org/10.1016/0141-1136(92)90002-4.
117. Martínez Ribes, L.; Basterretxea, G.; Palmer, M.; Tintoré, J. Origin and abundance of beach debris in the Balearic Islands. *Sci. Mar.* **2007**, *71*, 305–314.
118. Prevenios, M.; Zeri, C.; Tsangaris, C.; Liubartseva, S.; Fakiris, E.; Papatheodorou, G. Beach litter dynamics on Mediterranean coasts: Distinguishing sources and pathways. *Mar. Pollut. Bull.* **2018**, *129*, 448–457. https://doi.org/10.1016/j.marpolbul.2017.10.013.
119. Cesarini, G.; Cera, A.; Battisti, C.; Taurozzi, D.; Scalici, M. Is the weight of plastic litter correlated with vegetal wrack? A case study from a Central Italian beach. *Mar. Pollut. Bull.* **2021**, *171*, 112794. https://doi.org/10.1016/j.marpolbul.2021.112794.

120. Cresta, E.; Battisti, C. Anthropogenic litter along a coastal-wetland gradient: Reed-bed vegetation in the backdunes may act as a sink for expanded polystyrene. *Mar. Pollut. Bull.* **2021**, *172*, 112829. https://doi.org/10.1016/j.marpolbul.2021.112829.
122. Battisti, C.; Poeta, G.; Pietrelli, L.; Acosta, A.T.R. An Unexpected Consequence of Plastic Litter Clean-Up on Beaches: Too Much Sand Might Be Removed. *Environ. Pract.* **2016**, *18*, 242–246. https://doi.org/10.1017/S1466046616000417.
123. Maziane, F.; Nachite, D.; Anfuso, G. Artificial polymer materials debris characteristics along the Moroccan Mediterranean coast. *Mar. Pollut. Bull.* **2018**, *128*, 1–7. https://doi.org/10.1016/j.marpolbul.2017.12.067.
124. Grelaud, M.; Ziveri, P. The generation of marine litter in Mediterranean island beaches as an effect of tourism and its mitigation. *Sci. Rep.* **2020**, *10*, 20326. https://doi.org/10.1038/s41598-020-77225-5.
126. Poeta, G.; Battisti, C.; Bazzichetto, M.; Acosta, A.T.R. The cotton buds beach: Marine litter assessment along the Tyrrhenian coast of central Italy following the marine strategy framework directive criteria. *Mar. Pollut. Bull.* **2016**, *113*, 266–270. https://doi.org/10.1016/j.marpolbul.2016.09.035.
127. Gündoğdu, S.; Çevik, C. Mediterranean dirty edge: High level of meso and macroplastics pollution on the Turkish coast. *Environ. Pollut.* **2019**, *255*, 113351. https://doi.org/10.1016/j.envpol.2019.113351.
131. de Francesco, M.C.; Carranza, M.L.; Stanisci, A. Beach litter in Mediterranean coastal dunes: An insight on the Adriatic coast (central Italy). *Rend. Lincei. Sci. Fis. E Nat.* **2018**, *29*, 825–830. https://doi.org/10.1007/s12210-018-0740-5.
135. Battisti, C.; Fanelli, G.; Filpa, A.; Cerfolli, F. Giant Reed (Arundo donax) wrack as sink for plastic beach litter: First evidence and implication. *Mar. Pollut. Bull.* **2020**, *155*, 111179. https://doi.org/10.1016/j.marpolbul.2020.111179.
136. Menicagli, V.; Balestri, E.; Fulignati, S.; Galletti, A.M.R.; Lardicci, C. Plastic litter in coastal sand dunes: Degradation behavior and impact on native and non-native invasive plants. *Environ. Pollut.* **2023**, *316*, 120738. https://doi.org/10.1016/j.envpol.2022.120738.
140. Kordella, S.; Geraga, M.; Papatheodorou, G.; Fakiris, E.; Mitropoulou, I.M. Litter composition and source contribution for 80 beaches in Greece, Eastern Mediterranean: A nationwide voluntary clean-up campaign. *Aquat. Ecosyst. Health* **2013**, *16*, 111-118. https://doi.org/10.1080/14634988.2012.759503.
141. Gönülal, O.; Öz, İ.; Güreşen, S.O.; Öztürk, B. Abundance and composition of marine litter around Gökçeada Island (Northern Aegean Sea). *Aquat. Ecosyst. Health Manag.* **2016**, *19*, 461-467. https://doi.org/10.1080/14634988.2016.1257898.
142. Alshawafi, A.; Analla, M.; Alwashali, E; Aksissou, M. Assessment of marine debris on the coastal wetland of Martil in the North-East of Morocco. *Mar. Pollut. Bull.* **2017**, *117*, 302-310. https://doi.org/10.1016/j.marpolbul.2017.01.079.
143. Lots, F.A.E.; Behrens, P.; Vijver, M.G.; Horton, A.A.; Bosker, T. A large-scale investigation of microplastic contamination: Abundance and characteristics of microplastics in European beach sediment. *Mar. Pollut. Bull.* **2017**, *123*, 219-226. https://doi.org/10.1016/j.marpolbul.2017.08.057.
144. Munari, C.; Scoponi, M.; Mistri, M. Plastic debris in the Mediterranean Sea: types, occurrence and distribution along Adriatic shorelines. *Waste Manag.* **2017**, *67*, 385-391. https://doi.org/10.1016/j.wasman.2017.05.020.
145. Pasternak, G.; Zviely, D.; Ribic, C.A.; Ariel, A.; Spanier, E. Sources, composition and spatial distribution of marine debris along the Mediterranean coast of Israel. *Mar. Pollut. Bull.* **2017**, *114*, 1036-1045. https://doi.org/10.1016/j.marpolbul.2016.11.023.
146. Portman, M.E.; Brennan, R.E. Marine litter from beach-based sources: Case study of an Eastern Mediterranean coastal town. *Waste Manag.* **2017**, *69*, 535-544. https://doi.org/10.1016/j.wasman.2017.07.040.
147. Zhang, H. Transport of microplastics in coastal seas. *Estuar. Coast. Shelf Sci.* **2017**, *199*, 74-86. https://doi.org/10.1016/j.ecss.2017.09.032.
148. Duncan, E.M.; Arrowsmith, J.; Bain, C., Broderick, A.C.; Lee, J.; Metcalfe, K.; Pikesley, S.K.; Snape, R.T.E.; van Sebille, E.; Godley, B.J. The true depth of the Mediterranean plastic problem: Extreme microplastic pollution on marine turtle nesting beaches in Cyprus. *Mar. Pollut. Bull.* **2018**, *136*, 334-340. https://doi.org/10.1016/j.marpolbul.2018.09.019.

149. Karkanorachaki, K.; Kiparissis, S.; Kalogerakis, G.C.; Yiantzi, E.; Psillakis, E.; Kalogerakis, N. Plastic pellets, meso-and microplastics on the coastline of Northern Crete: Distribution and organic pollution. *Mar. Pollut. Bull.* **2018**, *133*, 578-589. https://doi.org/10.1016/j.marpolbul.2018.06.011.
150. Loizidou, X.I.; Loizides, M.I.; Orthodoxou, D.L. Persistent marine litter: small plastics and cigarette butts remain on beaches after organized beach cleanups. *Environ. Monit. Assess.* **2018**, *190*, 414. https://doi.org/10.1007/s10661-018-6798-9.
151. Vlachogianni, T.; Fortibuoni, T.; Ronchi, F.; Zeri, C.; Mazziotti, C.; Tutman, P.; Varezić, D.B.; Palatinus, A.; Trdan, Š.; Peterlin, M.; et al. Marine litter on the beaches of the Adriatic and Ionian Seas: An assessment of their abundance, composition and sources. *Mar. Pollut. Bull.* **2018**, *131*, 745-756. https://doi.org/10.1016/j.marpolbul.2018.05.006.
152. Asensio-Montesinos, F.; Anfuso, G.; Randerson, P.; Williams, A.T. Seasonal comparison of beach litter on Mediterranean coastal sites (Alicante, SE Spain). *Ocean Coast. Manag.* **2019**, *181*, 104914. https://doi.org/10.1016/j.ocecoaman.2019.104914.
153. Asensio-Montesinos, F.; Anfuso, G.; Williams, A.T. Beach litter distribution along the western Mediterranean coast of Spain. *Mar. Pollut. Bull.* **2019**, *141*, 119-126. https://doi.org/10.1016/j.marpolbul.2019.02.031.
154. Battisti, C.; Kroha, S.; Kozhuharova, E.; De Michelis, S.; Fanelli, G.; Poeta, G.; Pietrelli, L.; Cerfolli, F. Fishing lines and fishhooks as neglected marine litter: first data on chemical composition, densities, and biological entrapment from a Mediterranean beach. Environ. *Sci. Pollut. Res.* **2019**, *26*, 1000-1007. https://doi.org/10.1007/s11356-018-3753-9.
155. Constant, M.; Kerhervé, P.; Mino-Vercellio-Verollet, M.; Dumontier, M.; Sànchez Vidal, A.; Canals, M.; Heussner, S. Beached microplastics in the Northwestern Mediterranean Sea. *Mar. Pollut. Bull.* **2019**, *142*, 263-273. https://doi.org/10.1016/j.marpolbul.2019.03.032.
156. Mokos, M.; Zamora Martinez, I.; Zubak, I. Is central Croatian Adriatic Sea under plastic attack? Preliminary results of composition, abundance and sources of marine litter on three beaches. Rend. Fis. Acc. Lincei 2019, 30, 797-806. https://doi.org/10.1007/s12210-019-00851-3.
157. Nachite, D.; Maziane, F.; Anfuso, G.; Williams, A.T. Spatial and temporal variations of litter at the Mediterranean beaches of Mo-rocco mainly due to beach users. *Ocean Coast.Manag.* **2019**, *179*, 104846. https://doi.org/10.1016/j.ocecoaman.2019.104846.
158. Lechthaler, S.; Waldschläger, K.; Stauch, G.; Schüttrumpf, H. The way of macroplastic through the environment. *Enviroments* **2020**, *7*, 73. https://doi.org/10.3390/environments7100073.
159. Mokos, M.; Rokov, T.; Zubak Čižmek, I. Monitoring and analysis of marine litter in Vodenjak cove on Iž Island, central Croatian Adriatic Sea. *Rend. Fis. Acc. Lincei* **2020**, *31*, 905–912. https://doi.org/10.1007/s12210-020-00934-6.
160. Papachristopoulou, I.; Filippides, A.; Fakiris, E.; Papatheodorou, G. Vessel based photographic assessment of beach litter in remote coasts. A wide scale application in Saronikos Gulf, Greece. *Mar. Pollut. Bull.* **2020**, *150*, 110684. https://doi.org/10.1016/j.marpolbul.2019.110684.
161. Vlachogianni, T.; Skocir, M.; Constantin, P.; Labbe, C.; Orthodoxou, D.; Pesmatzoglou, I.; Scannella, D.; Spika, M.; Zissimopoulos, V.; Scoullos, M. Plastic pollution on the Mediter-ranean coastline: generating fit-for-purpose data to support decisionmak-ing via a participatory-science initiative. *Sci. Total Environ.* **2020**, *711*, 135058. https://doi.org/10.1016/j.scitotenv.2019.135058.
162. Asensio-Montesinos, F.; Anfuso, G.; Aguilar-Torrelo, M.T.; Oliva Ramirez, M. Abundance and temporal distribution of beach litter on the coast of Ceuta (North Africa, Gibraltar Strait). *Water* **2021**, *13*, 2739. https://doi.org/10.3390/w13192739.
163. Asensio-Montesinos, F.; Anfuso, G.; Williams, A.T.; Sanz-Lazaro, C. Litter behaviour on Mediterranean cobble beaches, SE Spain. *Mar. Pollut. Bull.* **2021**, *173*, 113106. https://doi.org/10.1016/j.marpolbul.2021.113106.
164. Benaissa, N.; Haddad, F.Z.; Taleb Bendiab, A.A.; Bensahla-Talet, L.; Hamou, A.; Dimache, A. A preliminary study on mac-ro-litter pollution on beaches along Aïn El Turk Bay. *Geoecomarina* **2021**, *27*, 83-89. https://doi.org/10.5281/5779786.

165. Bozzeda, F.; Zangaro, F.; Colangelo, M.A.; Pinna, M. Relationships between size and abundance in beach plastics: a power-law approach. *Mar. Pollut. Bull.* **2021**, *173*, 113005. https://doi.org/10.1016/j.marpolbul.2021.113005.
166. Camedda, A.; Coppa, S.; Palazzo, L.; Marra, S.; Massaro, G.; Serrentino, F.; Vencato, S.; Brundu, R.; de Lucia, G.A. Characterization and assessment of micro and macroscopic litter in sardinian beaches (Western Mediterranean Sea). *Water Air Soil Pollut.* **2021**, *232*, 1-14. https://doi.org/10.1007/s11270-021-04993-9.
167. Expósito, N.; Rovira, J.; Sierra, J.; Folch, J.; Schuhmacher, M. Microplastics levels, size, morphology and composition in marine water, sediments and sand beaches. Case study of Tarragona coast (western Mediterranean). *Sci. Total Environ.* **2021**, *786*, 147453. https://doi.org/10.1016/j.scitotenv.2021.147453.
168. Fanini, L.; Guittard, A. On single use plastic straws: pre-ban findings on touristic beaches in Crete. *Mar. Pollut. Bull.* **2021**, *171*, 112790. https://doi.org/10.1016/j.marpolbul.2021.112790.
169. Fortibuoni, T.; Amadesi, N.; Vlachogianni, T. Composition and abundance of macrolitter along the Italian coastline: The first baseline assessment within the european Marine Strategy Framework Directive. *Environ. Pollut.* **2021**, *268*, 115886. https://doi.org/10.1016/j.envpol.2020.115886.
170. Frigione, M.; Marini, G.; Pinna, M. A thermal analysis-based approach to identify different waste macroplastics in beach litter: The case study of aquatina di frigole natura 2000 site (it9150003, Italy). *Sustainability* **2021**, *13*, 3186. https://doi.org/10.3390/su13063186.
171. Mandić, M.; Gvozdenović, S.; Peraš, I.; Ivanović, A.; Malovrazić, N. Quantification and classification of beach litter in Montenegro (South-East Adriatic Sea). In: *Handbook of Environmental Chemistry*. Springer Science and Business Media Deutschland GmbH, University of Montenegro, Institute of Marine Biology, Kotor, Montenegro, 2021. pp. 257–274. https://doi.org/10.1007/698_2020_715.
172. Martín-Lara, M.A.; Godoy, V.; Quesada, L.; Lozano, E.J.; Calero, M. Environmental status of marine plastic pollution in Spain. *Mar. Pollut. Bull.* **2021**, *170*, 112677. https://doi.org/10.1016/j.marpolbul.2021.112677.
173. Merlino, S.; Paterni, M.; Locritani, M.; Andriolo, U.; Gonçalves, G.; Massetti, L. Citizen science for marine litter detection and classification on unmanned aerial vehicle images. *Water* **2021**, *13*, 3349. https://doi.org/10.3390/w13233349.
174. Özden, Ö.; Yıldırım, S.; Fuller, W.J.; Godley, B.J. "Anthropogenic marine litter on the north coast of Cyprus: insights into marine pollution in the eastern Mediterranean marine pollution in the eastern Mediterranean". *Mar. Pollut. Bull.* **2021**, *165*, 112167. https://doi.org/10.1016/j.marpolbul.2021.112167.
175. Phuong, N.N.; Fauvelle, V.; Grenz, C.; Ourgaud, M.; Schmidt, N.; Strady, E.; Sempéré, R. Highlights from a review of microplastics in marine sediments. *Sci. Total Environ.* **2021**, *777*, 146225. https://doi.org/10.1016/j.scitotenv.2021.146225.
176. Romiti, F.; Pietrangeli, E.; Battisti, C.; Carpaneto, G.M. Quantifying the entrapment effect of anthropogenic beach litter on sand-dwelling beetles according to the EU Marine Strategy Framework Directive. *J. Insect Conserv.* **2021**, *25*, 441-452. https://doi.org/10.1007/s10841-021-00312-z.
177. Schmid, C.; Cozzarini, L.; Zambello, E. A critical review on marine litter in the Adriatic Sea: Focus on plastic pollution. *Environ. Pollut.* **2021**, *273*, 116430. https://doi.org/10.1016/j.envpol.2021.116430.
178. Schmid, C.; Cozzarini, L.; Zambello, E. Microplastic's story. *Mar. Pollut. Bull.* **2021**, *162*, 111820. https://doi.org/10.1016/j.marpolbul.2020.111820.121. Shiber, J.G.; Barrales-Rienda, J.M. Plastic pellets, tar, and megalitter on Beirut beaches, 1977–1988. *Environ. Pollut.* **1991**, *71*, 17–30. https://doi.org/10.1016/0269-7491(91)90041-T.
179. Taïbi, N.-E.; El Amine Bentaallah, M.; Alomar, C.; Compa, M.; Deudero, S. Micro- and macro-plastics in beach sediment of the Algerian western coast: first data on distribution, characterization, and source. *Mar. Pollut. Bull.* **2021**, *165*, 112168. https://doi.org/10.1016/j.marpolbul.2021.112168.
180. Bonanno, G. Marine-protected areas and plastic pollution. In: *Plastic Pollution and Marine Conservation: Approaches to Protect Biodiversity and Marine Life*. Academic Press, 2022. pp. 249–273. https://doi.org/10.1016/B978-0-12-822471-7.00010-9.

181. Cesarini, G.; Secco, S.; Battisti, C.; Questino, B.; Marcello, L.; Scalici, M. Temporal changes of plastic litter and associated encrusting biota: Evidence from Central Italy (Mediterranean Sea). *Mar. Pollut. Bull.* **2022**, *181*, 113890. https://doi.org/10.1016/j.marpolbul.2022.113890.
182. Corbau, C.; Lazarou, A.; Gazale, V.; Nardin, W.; Simeoni, U.; Carboni, D. What can beach litter tell about local management: A comparison of five pocket beaches of the North Sardinia island (Italy). *Mar. Pollut. Bull.* **2022**, *174*, 113170. https://doi.org/10.1016/j.marpolbul.2021.113170.
183. Menicagli, V.; De Battisti, D.; Balestri, E.; Federigi, I.; Maltagliati, F.; Verani, M.; Castelli, A.; Carducci, A.; Lardicci, C. Impact of storms and proximity to entry points on marine litter and wrack accumulation along Mediterranean beaches: Management implications. *Sci. Total Environ.* **2022**, *824*, 153914. https://doi.org/10.1016/j.scitotenv.2022.153914.
184. Nithin, A.; Sundaramanickam, A.; Bratovcic, A.; Surya, P.; Sathish, M. Microplastics Occurrence in Different Regions Around the World. In: *Microplastics Pollution in Aquatic Media. Environmental Footprints and Eco-design of Products and Processes*. Springer, Singapore, 2022. https://doi.org/10.1007/978-981-16-8440-1_1.
185. Novillo-Sanjuan, O.; Raga, J.A.; Tomás, J. Microdebris in three Spanish Mediterranean beaches located at a sporadic loggerhead turtles' (Caretta caretta) nesting area. *Reg. Stud. Mar. Sci.* **2022**, *49*, 102116. https://doi.org/10.1016/j.rsma.2021.102116.
186. Orthodoxou, D.L.; Loizidou, X.I.; Baldwin, C.; Kocareis, C.; Karonias, A.; Ateş, M.A. Seasonal and geographic variations of marine litter: A comprehensive study from the island of Cyprus. *Mar. Pollut. Bull.* **2022**, *177*, 113495. https://doi.org/10.1016/j.marpolbul.2022.113495.
187. Perumal, K.; Muthuramalingam, S. Global sources, abundance, size, and distribution of microplastics in marine sediments - A critical review. *Estuar. Coast. Shelf Sci.* **2022**, *264*, 107702. https://doi.org/10.1016/j.ecss.2021.107702.
188. Poeta, G.; Bazzichetto, M.; Gallitelli, L.; Garzia, M.; Aprea, F.; Bartoli, F.; Battisti, C.; Cascone, S.; Corradi, A.; D'Amelia, D.; et al. One year after on Tyrrhenian coasts: The ban of cotton buds does not reduce their dominance in beach litter composition. *Mar. Policy* **2022**, *143*, 105195. https://doi.org/10.1016/j.marpol.2022.105195.
189. Prabhu, P.P.; Pan, K.; Krishnan, J.N. Microplastics: Global occurrence, impact, characteristics and sorting. *Front. Mar. Sci.* **2022**, *9*, 893641. https://doi.org/10.3389/fmars.2022.893641.
190. Rubin, A.E.; Omeysi, L.; Zucker, I. Mediterranean microplastic contamination: Israel's coastline contributions. *Mar. Pollut. Bull.* **2022**, *183*, 114080. https://doi.org/10.1016/j.marpolbul.2022.114080.
191. Simon-Sánchez, L.; Grelaud, M.; Franci, M.; Ziveri, P. Are research methods shaping our understanding of microplastic pollution? A literature review on the seawater and sediment bodies of the Mediterranean Sea. *Environ. Pollut.* **2022**, *292*, 118275. https://doi.org/10.1016/j.envpol.2021.118275.
192. Strafella, P.; López Correa, M.; Pyko, I.; Teichert, S.; Gomiero, A. Distribution of Microplastics in the Marine Environment. In: *Handbook of Microplastics in the Environment*. Springer, Cham, 2022. https://doi.org/10.1007/978-3-030-39041-9_43.
193. Celine, M.; Sharif, J.; Maria, K.; El Rahman, H.A.; Myriam, L.; Myriam, G.; Anthony, O.; Rachid, A.; Milad, F. First assessment of microplastics in offshore sediments along the Lebanese coast, South-Eastern Mediterranean. *Mar. Pollut. Bull.* **2023**, *186*, 114422. https://doi.org/10.1016/j.marpolbul.2022.114422.
194. Fagiano, V.; Compa, M.; Alomar, C.; Rios-Fuster, B.; Morató, M.; Capó, X.; Deudero, S. Breaking the paradigm: Marine sediments hold two-fold microplastics than sea surface waters and are dominated by fibers. *Sci. Total Environ.* **2023**, *858*, 159722. https://doi.org/10.1016/j.scitotenv.2022.159722.
195. John, K.I.; Omorogie, M.O.; Adeleye, A.T.; Bayode, A.A.; Helmreich, B. Environmental Microplastics Distribution, Impact, and Determination Methods: a Review. *J. Anal. Chem.* **2023**, *78*, 1199-1212. https://doi.org/10.1134/S106193482309006X.

196. Ma, Y.-B.; Xie, Z.-Y.; Hamid, N.; Tang, Q.-P.; Deng, J.-Y.; Luo, L.; Pei, D.-S. Recent advances in micro (nano) plastics in the environment: Distribution, health risks, challenges and future prospects. *Aquat. Toxicol.* **2023**, *261*, 106597. https://doi.org/10.1016/j.aquatox.2023.106597.
197. Malli, A.; Shehayeb, A.; Yehya, A. Occurrence and risks of microplastics in the ecosystems of the Middle East and North Africa (MENA). *Environ. Sci. Pollut. Res.* **2023**, *30*, 64800–64826. https://doi.org/10.1007/s11356-023-27029-7.
198. Mghili, B.; Imane, L.; Bouzekry, A.; Gunasekaran, K.; Aksissou, M. Cigarette butt pollution in popular beaches of Morocco: Abundance, distribution, and mitigation measures. *Mar. Pollut. Bull.* **2023**, *195*, 115530. https://doi.org/10.1016/j.marpolbul.2023.115530.
199. Noureddine, E-R.; Nachite, D.; Anfuso, G.; Azaaouaj, S. The Sector Analysis as a Coastal Management Tool for Sustainable Tourism Development on the Mediterranean Coast of Morocco. *Sustainability* **2023**, *15*, 12581. https://doi.org/10.3390/su151612581.
200. Rios-Fuster, B.; Compa, M.; Alomar, C.; Deudero, S. Stranded and floating marine debris detected along the coastline of Cabrera National Park (Balearic Islands). *Mar. Pollut. Bull.* **2023**, 194, 115288. https://doi.org/10.1016/j.marpolbul.2023.115288.
201. Vlachogianni, T.; Scoullos, M. Assessing marine macrolitter on the coastline of the Asterousia Biosphere Reserve: Insights from a community-based study. *Mar. Pollut. Bull.* **2023**, *195*, 115474. https://doi.org/10.1016/j.marpolbul.2023.115474.
